# The forest beyond the trees: A network perspective on governing co-production of nature’s contributions to people

**DOI:** 10.1007/s13280-025-02187-9

**Published:** 2025-04-26

**Authors:** Roman Isaac, Graeme S. Cumming, María R. Felipe-Lucia, Berta Martín-López

**Affiliations:** 1 Social-Ecological Systems Institute, Leuphana University of Lüneburg, Universitätsallee 1, 21335 Lüneburg, Germany; 2 Oceans Institute, The University of Western Australia (M053), 35 Stirling Highway, Perth, 6009 Australia; 3 CSIC-Instituto Pirenaico de Ecología (IPE, CSIC), Av. Nuestra Señora de la Victoria 16, 22700 Jaca, Huesca Spain

**Keywords:** Anthropogenic assets, Ecosystem services co-production, Forest management, Sustainable management

## Abstract

Nature’s contributions to people (NCP) provided by forests are co-produced by an interplay of natural and anthropogenic capitals, including human, social, physical, and financial capital, which are influenced by various actors across multiple levels. Here, we assessed the co-production of four forest NCP (timber, habitat creation and maintenance, climate regulation, non-material NCP). We conducted social network analyses based on interviews with local forest actors to (i) understand which actors are most relevant for managing different anthropogenic capitals in NCP co-production and (ii) identify patterns of governing co-produced forest NCP via actor relationships. Our findings revealed three patterns: (1) *governance of timber production *via* financial flows*; (2) *governance of knowledge and labour for climate regulation*; and (3) *governance for habitat management*. Making actor–capital relationships tangible provides evidence to inform decision-making by tracing how specific actors favour certain capitals potentially influencing the sustainability of NCP co-production.

**Supplementary Information:**

The online version contains supplementary material available at 10.1007/s13280-025-02187-9.

## Introduction

Forest ecosystems around the world are coming under increasing pressure from human demands, shifting weather patterns, and land-use changes (Allen et al. 2015; Haddad et al. 2015; Brodribb et al. 2020). Responding proactively to these pressures will require flexible governance and management systems that facilitate rapid responses to ensure the long-term provision of and access to nature’s contributions to people (NCP) provided by forest ecosystems (Hernández-Morcillo et al. 2022; Felton et al. 2023). Although there has been considerable research on adapting to these challenges by individuals or organisations (e.g. Bolte et al. 2009; Keskitalo 2013; Hernández-Morcillo et al. 2022; Mann et al. 2022), adaptation across more diverse actor networks remains poorly understood. Understanding how social networks composed of different actors perceive and respond to changes in forest ecosystems to balance trade-offs between different forest NCP is a first step towards the requisite network management strategies.

In forests and other ecosystems, NCP are produced by an interplay between nature and humans in a process known as co-production (Spangenberg et al. 2014; Lavorel et al. 2020), which relies on various anthropogenic capitals: human, social, physical, and financial (Palomo et al. 2016; Kachler et al. 2023). People modify and manage forests to supply NCP, and forests respond to the actions of humans through ecological dynamics such as growth, herbivory, and succession (Reyers et al. 2013; Bruley et al. 2021). In a forest context, anthropogenic capitals refer to knowledge and skills within forest management practices or manual labour needed to harvest timber (human capital); rules and norms, such as forest certifications (social capital); machinery and tools, such as chainsaws or harvesters, and infrastructure, such as skid roads, (physical capital); and monetary stocks and flows, including governmental direct payments, loans, or savings (financial capital) (see S1 for detailed definitions) (Palomo et al. 2016; Isaac et al. 2024b).

The co-production concept has been applied across various ecosystems, including forests (Torralba et al. 2018; e.g. Bruley et al. 2021; Palliwoda et al. 2021), to understand the relative importance of natural and anthropogenic assets, the substitutability or reversibility of capital use, and the sustainability of NCP co-production (Locatelli et al. 2024). Management approaches to the co-production of forest NCP materialise in multiple forms. For example, timber may be harvested using suitable machinery or tools (Fischer and Eastwood 2016); agroforestry practices can support micro-climate regulation by increasing shade via tree cover expansion (Lavorel et al. 2020); and pruning or tree lopping, which are based on the use of tools and manual labour, may lead not only to more expansive tree cover but also to higher acorn production, a fruit traditionally used as fodder around the Mediterranean (Garrido et al. 2017). Similarly, locally adapted forest management may draw on manual labour, knowledge, and husbandry skills to co-produce numerous regulating NCP. For example, afforestation of high-alpine slopes may mitigate the risk of avalanches and create habitat for species (Bruley et al. 2021); and traditional burning practices in Australian woodlands can reduce the likelihood of destructive hot fires (Yibarbuk et al. 2001). Understanding how anthropogenic capitals are used to co-produce NCP, and by whom, is crucial for land management decisions that aim for the sustainable provision and use of NCP (Rieb et al. 2023; Locatelli et al. 2024).

Forest managers’ and owners’ decisions influence which NCP are prioritised (Mann et al. 2022) and how they are co-produced. For example, multi-functional, integrated, or multi-objective forest management supports the provision of multiple forest NCP, while intensive forestry management approaches predominantly focus on the production of timber (Pukkala 2016; Borrass et al. 2017). Decisions related to forest management and the prioritisation of NCP and mobilisation of anthropogenic capitals rely on processes established by diverse actors in formal and informal, often polycentric, governance settings across multiple levels (e.g. Arts 2021; Primmer et al. 2021; Mann et al. 2022). Forest governance typically includes “network-like arrangements of public and private actors, self-regulation by market organisations, public–private partnerships, emission trading schemes, covenants, certification programs, etc.” (Arts and Buizer 2009, 344). Thus, actor relationships in formal and informal networks can shape how forests and their contributions to people are managed (e.g. Korhonen et al. 2012; Keskitalo et al. 2014; Schulz et al. 2018). For example, in Switzerland, where forest policy-making is mainly driven by a small network of expert knowledge holders, Schulz et al. (2018) demonstrated a significant effect of non-governmental organisation (NGO) networks on forest policy. Actor networks also promote trust and information exchanges, which have been described as enabling factors for collaboration in NCP and forest governance across governance levels, from local to international, and across sectors (e.g. Keskitalo et al. 2014; Borg et al. 2015; Stoettner and Ní Dhubháin 2019). Despite the relevant role of actor networks in shaping forest governance processes (e.g. Korhonen et al. 2012; Keskitalo et al. 2014; Knoot and Rickenbach 2014; Borg et al. 2015), social network effects on the governance of NCP co-production, in particular the effects on anthropogenic capital use, have only recently been investigated in few studies (e.g. Jericó-Daminello et al. 2021; Barraclough et al. 2022).

Previously, the influence of forest governance networks on NCP co-production has been studied only implicitly. For example, by exploring the interlinkages between social capital and governance of forests resources (Górriz-Mifsud et al. 2016) or investigating how network interactions underpin the establishment of mushroom hunting regulations (Górriz-Mifsud et al. 2017). Despite these studies, it remains uncertain how actors are connected across governance levels via specific anthropogenic capitals underpinning forest NCP.

To address this research gap and understand how local forest actors perceive the governance of anthropogenic capitals in forest NCP co-production, we conducted social network analyses (SNA) based on interviews in three case study sites across Germany. Specifically, we aimed to identify (i) which actors were most relevant for the governance of specific anthropogenic capitals in forest NCP co-production; and (ii) patterns of actors and their connections via anthropogenic capitals when governing forest NCP co-production.

## Materials and methods

### Study sites

**Fig. 1:**
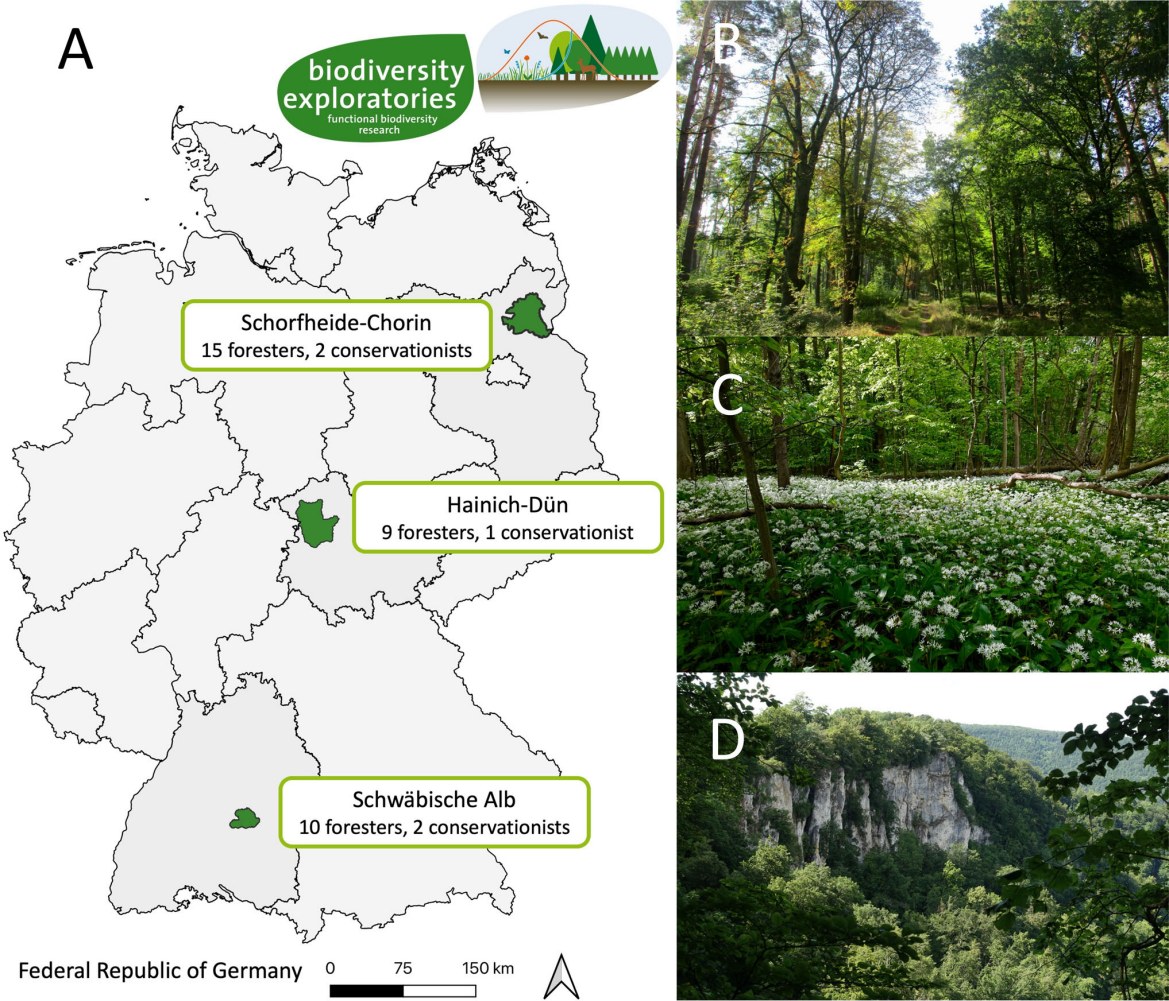
**A** Map of the case study sites in Germany indicating the number of interviews per case study site and sector the respondents belong to (total number of interviews *n* = 39). This figure is based on data provided by the Biodiversity Exploratories Information System (BExIS) (2022) and Esri Deutschland GmbH (2018). Photos of forests in the case study sites: **B** Schorfheide-Chorin (photo by Berta Martín-López), **C** Hainich-Dün, **D** Schwäbische Alb (both photos by Roman Isaac)

This research is embedded in the long-term and large-scale research platform *Biodiversity Exploratories* and focuses on three study sites across Germany, located in the Schorfheide-Chorin (State of Brandenburg) in the North East, the Hainich-Dün (Free State of Thuringia) in the centre, and the Schwäbische Alb (State of Baden-Württemberg) in the South West (Fischer et al. 2010b) (Fig. 1). Schorfheide-Chorin encompasses a UNESCO^1^ biosphere reserve, the World Heritage site *Grumsin Forest,* and is characterised by a diverse landscape of forests, lakes, and agricultural fields (Landesamt für Umwelt Brandenburg 2023; UNESCO 2023). Hainich-Dün is characterised by a mix of productive forests and agricultural land and contains the Hainich National Park, including another UNESCO World Heritage site (Nationalpark Hainich 2023; UNESCO 2023). Schwäbische Alb includes a UNESCO biosphere reserve and is characterised by forests and grasslands on a sub-montane and montane plateau (Biosphärengebiet Schwäbische Alb 2023).

### Data collection

Following other SNA studies (e.g. Knoot and Rickenbach 2014), we used interviews with forest actors as the basis for our analysis. We conducted 39 semi-structured interviews (17 in Schorfheide-Chorin, 10 in Hainich Dün, 12 in Schwäbische Alb) between May and November 2021 with local forest actors (33 foresters, one forestry contractor, two representatives for each biosphere reserve, and one representative for Hainich National Park) (Fig. 1). The interviews were pre-tested outside the study sites and adapted accordingly before being conducted. Most respondents were male (*n* = 37) with an average age of 53.5 years. We selected respondents in three ways. First, by approaching foresters within the *Biodiversity Exploratories’* experimental plots to participate. Second, by contacting the biosphere reserve and national park representatives via their respective administrations. Third and due to COVID-19 travel restrictions and the reluctance of some foresters to be interviewed, we followed an extended egocentric network approach by including nodes connected to direct contacts of an individual actor, i.e. “friends of friends” (Marin and Wellman 2011, p.20), an approach termed “snowball sampling”. Snowball sampling is a common technique when collecting data for social networks on NCP (Oteros-Rozas et al. 2014; e.g. Aguilar Rodríguez et al. 2021) and the willingness of potential respondents to participate is often higher when referred to by one of their friends or colleagues (Borgatti et al. 2022).

To begin the interview, we presented 11 NCP to the respondents using laminated images, each representing one NCP, and a whiteboard with magnets (Isaac et al. 2024a; Kachler et al. 2024). Respondents selected the five most relevant NCP for their well-being out of these 11 NCP. Subsequently respondents ranked the five selected NCP according to their importance to them, from least important to most important. We then asked a set of questions solely for the first-ranked NCP. We did so to avoid respondent fatigue due to lengthy interviews. Questions targeted the importance of the NCP, the use of different anthropogenic capitals in the management and use of the NCP, and how other actors or organisations influence the co-production of this specific NCP. In contrast to representatives of the national park and biosphere reserves, foresters answered additional and more specific questions regarding their management practices for timber production and habitat management to better understand the on the ground use of anthropogenic capitals. The interviews were recorded and anonymised before processing with the respondents’ consent and according to the approval of UNIVERSITY’S ethics review committee (EB-Antrag 2021-03-0-Martín-López). All interviews were transcribed using the NVIVO software (Alfasoft GmbH 2023).

### Data coding

The interview transcripts were analysed using a predefined coding set assessing the relationships between actor relevant for the co-production of NCP. To code NCP, we followed the Intergovernmental Science-Policy Platform on Biodiversity and Ecosystem Services (IPBES) generalising perspective (Díaz et al. 2018) and regrouped the presented NCP into four NCP groups: (1) timber production, (2) climate regulation (including carbon sequestration and micro-climate regulation), (3) habitat creation and maintenance (including habitat maintenance, pollination, and natural pest control, as a more diverse habitat likely provides better conditions for species that provide pest control and pollination (Palliwoda et al. 2021)), and (4) non-material NCP (including all physical activities in nature, the observation and enjoyment of plants, animals, and landscapes). The relationship between actors involved in NCP co-production was characterised by the governance level at which they operate, the actor group they belong to, the NCP they co-produce, and the anthropogenic capitals by which two actors were connected when doing so (Table 1; Table S2). These data were coded based on the description provided by the interviewees.

**Table 1:** Overview of codes used to assess the actor–actor relationship in forest NCP co-production characterised by the use of anthropogenic capitals. For full coding set, see Table S2

Category	Code	Subcode	Sources
Actor–actor relationship	Actor 1		Interviews
	Actor 2		Interviews
	Governance level of actors	Local (case study); federal state; German national; European Union	Isaac et al. (2024a, b)
	Actor group	Biodiversity Exploratories (project representatives and local management teams), foresters; forest industry; forest owners; government; protected areas; societal actors (i.e. NGOs, associations)	Interviews
Nature’s contribution to people (NCP)	Timber production		Díaz et al. (2018)
	Climate regulation	Carbon sequestration; micro-climate regulation	
	Habitat creation & maintenance	Habitat maintenance; pollination, natural pest control	
	Non-material NCP	Physical activities in nature; observation and enjoyment of plants animals, and landscape	
Anthropogenic capital	Human capital	Labour; knowledge; skills	Palomo et al. (2016)
	Social capital	Networks; institutions	
	Physical capital	Tools and machinery; infrastructure	
	Financial capital	Direct payments; subsidies; revenue	

### Social network analysis

**Fig. 2:**
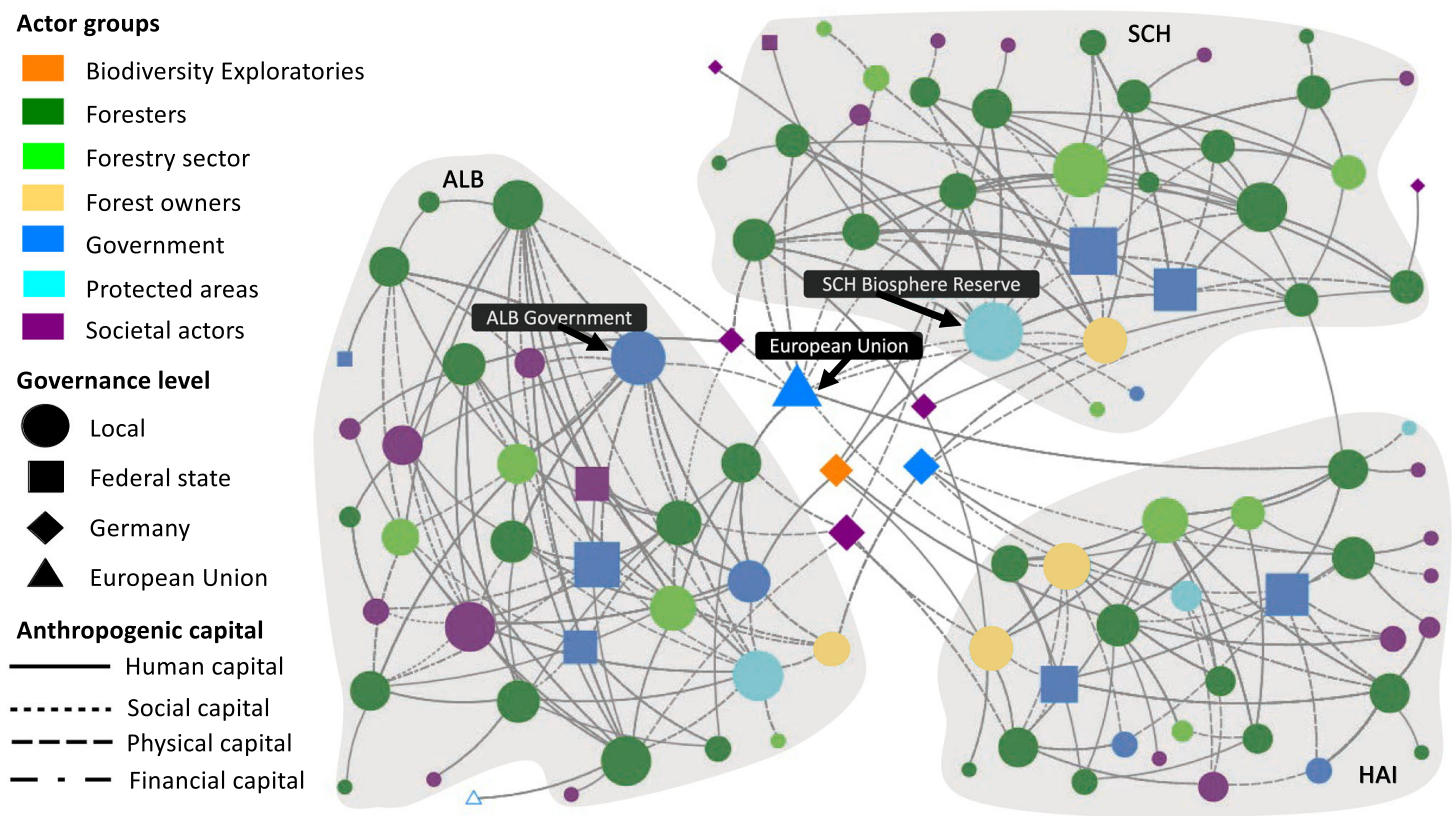
The combined network displaying the perceived connections between actors across multiple governance levels involved in the co-production of forest NCP, highlighting the three case study sites. Nodes portray actors: node size is proportional to actor’s degree centrality, node colours represent the actor group, node shapes represent the governance level at which this actor operates (local, federal state, German national, or EU level), and ties represent the anthropogenic capitals according to the legend. Tie strength is not considered. Grey underlay indicates actors in the same case study site (SCH = Schorfheide-Chorin, HAI = Hainich Dün, ALB = Schwäbische Alb) and respective federal state. The empty blue triangle represents the Austrian forest authority, which was the only actor not fitting to any of the governance levels described above

**Fig. 3:**
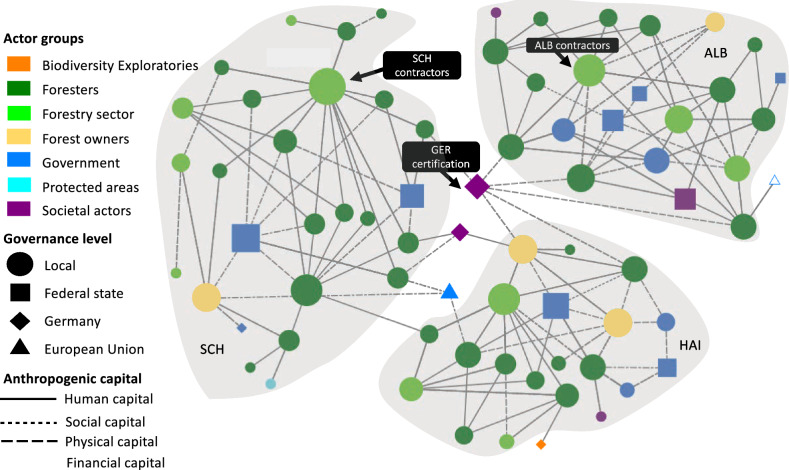
The network for timber production displays the perceived connections between actors across multiple governance levels involved in the co-production of timber. Nodes portray actors: node size is proportional to the actor’s degree centrality, node colours represent the actor group, node shapes represent the governance level at which this actor operates (local, federal state, German national, or EU level), and ties represent the anthropogenic capitals according to the legend. Tie strength is not considered. Grey underlay indicates actors in the same case study site (SCH = Schorfheide-Chorin, HAI = Hainich Dün, ALB = Schwäbische Alb) and respective federal state. The empty blue triangle represents the Austrian forest authority which was the only actor not fitting to any of the governance levels described above

**Fig. 4:**
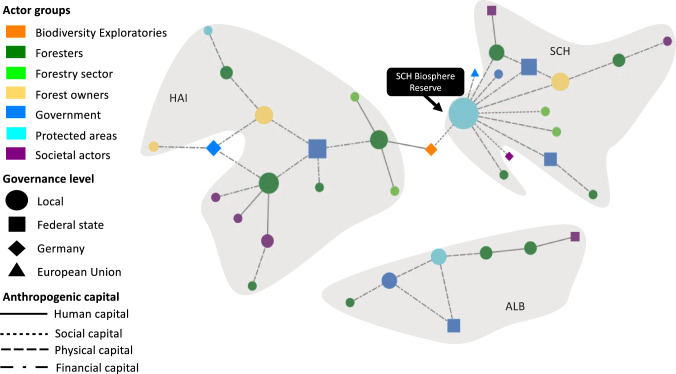
The network for the regulation of climate displays the perceived connections between actors across multiple governance levels contributing to climate regulation. Nodes portray actors: node size is proportional to the actor’s degree centrality, node colours represent the actor group, node shapes represent the governance level at which this actor operates (local, federal state, German national, or EU level), and ties represent the anthropogenic capitals according to the legend. Grey underlay indicates actors in the same case study site (SCH = Schorfheide-Chorin, HAI = Hainich Dün, ALB = Schwäbische Alb) and respective federal state

**Fig. 5:**
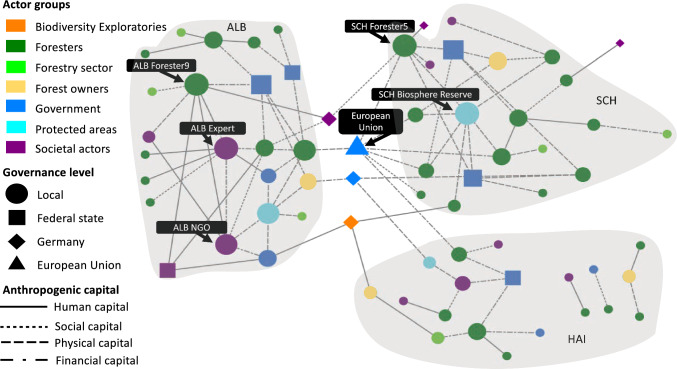
The network for habitat creation and maintenance displays the perceived connections between actors across multiple governance levels contributing to this NCP. Nodes portray actors: node size is proportional to the actor’s degree centrality based on degree, node colours represent the actor group, node shapes represent the governance level at which this actor operates (local, federal state, German national, or EU level), and ties represent the anthropogenic capitals according to the legend. Tie strength is not considered. Grey underlay indicates actors in the same case study site (SCH = Schorfheide-Chorin, HAI = Hainich Dün, ALB = Schwäbische Alb) and respective federal state

**Fig. 6:**
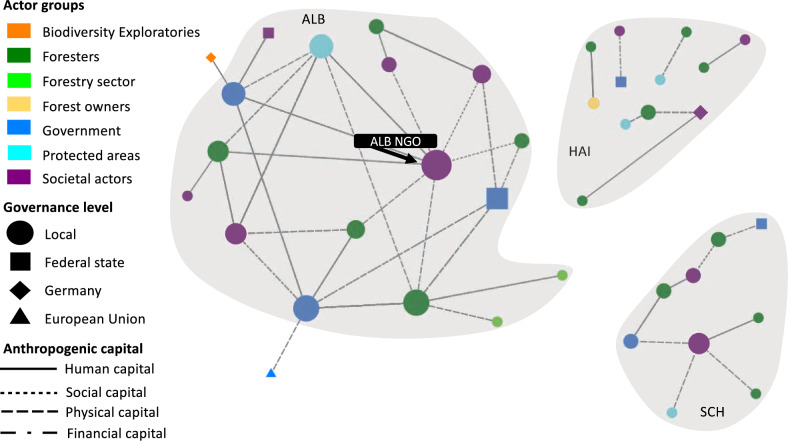
The network for non-material NCP displays the perceived connections between actors across multiple governance levels contributing to this NCP. Nodes portray actors: node size is proportional to the actor’s degree centrality, node colours represent the actor group, node shapes represent the governance level at which this actor operates (local, federal state, German national, or EU level), and ties represent the anthropogenic capitals according to the legend. Tie strength is not considered. Grey underlay indicates actors in the same case study site (SCH = Schorfheide-Chorin, HAI = Hainich Dün, ALB = Schwäbische Alb) and respective federal state

Social networks are formed by actors via one or multiple interactions (Marin and Wellman 2011). Actors within social networks are referred to as nodes, and their connections are known as ties (Borgatti et al. 2022). For a full description of social network analysis (SNA), see supplementary material S3. From the interview data, we extracted a list of ties between actors characterised by the anthropogenic capitals through which they were connected. Based on these ties, we generated and visualised a network for each NCP (timber production; climate regulation; habitat creation and maintenance; non-material NCP) plus a combined network based on these, using NodeXL and Gephi (Figs. 2, 3, 4, 5, and 6) (Bastian et al. 2009; Smith et al. 2010). The nodes within each network represent the actors identified by the respondents. The ties between the nodes represent the anthropogenic capitals used by the actors to co-produce forest NCP (see legend of Figs. 2, 3, 4, 5, and 6). For each node in each network, we calculated three commonly used centrality metrics: degree centrality, betweenness centrality, and eigenvector centrality (Hannemann and Riddle 2011; Borgatti et al. 2022) (see S3 for a full description). Further, we used a nonparametric Kruskal–Wallis test to identify whether differences for the centrality metrics existed among actor groups. We first tested for differences between the actor groups involved in the co-production of each focal NCP and the combined NCP network. We then tested whether connections between actor groups differed when using specific anthropogenic capital in the co-production of each NCP. When the Kruskal–Wallis test identified significant differences, we used Dunn’s pairwise comparison tests in both cases to evaluate differences between actor groups. All statistical analyses were performed in XLSTAT 2020.3.1, a plugin for Microsoft Excel (Addinsoft 2023).

### Methodological limitations

Our study is subject to several methodological limitations. First, we conducted research in three case study sites selected for the long-term and large-scale research platform *Biodiversity Exploratories* since they represent a gradient of forestry intensity (Fischer et al. 2010b). The two biosphere reserves and a national park within these sites may have influenced our results regarding the degree of collaboration between actors due to facilitation efforts by the respective protected area administrations. Second, snowball sampling may bias results as it favours the detection of highly connected actors (van der Hulst 2011). For example, this approach led to more interviews in Schorfheide-Chorin than in the other two study sites, which may have been an outcome of the high degree centrality found for the Biosphere Reserve administration in this site. Third, forests in our study are managed under the guiding principle “Schutz durch Nutzung” (“conservation through utilisation”) to foster the provision of multiple forest NCP (Borrass et al. 2017, 2), when in reality forest management still heavily relies on timber production as a source of income (Sotirov and Storch 2018; Lovrić et al. 2025). Thus, caution must be taken when generalising our findings since this guiding principle may have affected foresters’ perceptions regarding the impacts of their management for regulating NCP. In other words, the notion that managing forests for timber production is supporting regulating NCP may have led to denser networks for timber production and habitat creation and maintenance. Finally and in contrast to other countries, German forests and their management are strongly regulated by policy documents across multiple governance levels, limiting foresters in making decisions freely (Sotirov and Storch 2018; Isaac et al. 2024b). For example, some foresters in our study told us that highly productive parts of their forest were decommissioned to meet conservation targets set by federal state policy, making it more difficult to then provide the mass of timber set in their ten-year forest plan. Such instances may have influenced foresters’ statements regarding their management of regulating NCP.

## Results

### Governance networks affecting capitals in the co-production of forest NCP

Social network analysis identified four separate forest NCP networks and a combined network, including all NCP (Figs. 2, 3, 4, 5, and 6). For the combined network, the Biosphere Reserve administration in Schorfheide-Chorin (SCH Biosphere Reserve) had the highest degree centrality (Fig. 2, Table S4). The EU had the highest betweenness centrality since it facilitated most connections of otherwise unconnected actors. Local government on Schwäbische-Alb (ALB Government), i.e. municipalities and district authorities, had the highest eigenvector centrality, thus being most connected to other well-connected nodes (Fig. 2, Table S4). Between actor groups, we found highly significant differences for degree (*χ*^2^ = 19.696, d.f. = 6, *p* < 0.003) and betweenness (*χ*^2^ = 23.392, d.f. = 6, *p* < 0.001). Dunn’s multiple comparison test (*p* < 0.01) indicated that forest owners had the highest degree centrality while betweenness centrality was highest for the Biodiversity Exploratories and forest owners (Table S5).

### Timber production

For the network on timber production (Fig. 3), we found forestry contractors in Schorfheide-Chorin (SCH forestry contractors) to have the highest degree centrality. Forest certification (GER forest certification), referring to both the Forest Stewardship Council (FSC) and the Programme for the Endorsement of Forest Certification Schemes (PEFC), had the highest betweenness centrality, exerting high control over the network by providing rules for forest management (social capital) (Table S4). Forestry contractors in Schwäbische Alb (ALB Forestry contractors) scored highest for eigenvector centrality (Fig. 3). Here, we did not find significant differences between centrality metrics among the actor groups. However, we found differences in degree (*χ*^2^ = 7.728, d.f. = 3, *p* < 0.052) and betweenness centrality (*χ*^2^ = 7.068, d.f. = 3, *p* < 0.070) between actor groups regarding their influence on financial capital (Table 2). The forest owners group scored highest for degree and betweenness centrality (based on Dunn’s multiple comparison test (*p* < 0.01), Table S6. Here, statistical tests revealed a pattern showing the importance of forest owners for timber production by being connected to other actors via financial capital, referred to as *Pattern 1 governance of timber production *via* financial flows*.

**Table 2:** *P* values from the Kruskal–Wallis test for degree centrality, betweenness centrality, and eigenvector centrality showing differences between actor groups in their influence on the use of each anthropogenic capital underpinning the co-production of timber production, climate regulation, habitat creation and maintenance and non-material NCP. Significant *p* values are presented in bold font

Anthropogenic capital category	Network centrality metric	Nature’s contributions to people (NCP)			
		Timber production	Climate regulation	Habitat creation & maintenance	Non-material NCP
Human capital	Degree	0.115	0.169	0.384	0.399
	Betweenness	0.183	0.169	0.325	0.374
	Eigenvector	0.207	**0.053**	0.243	0.536
Social capital	Degree	0.253	0.394	**0.043**	0.799
	Betweenness	0.295	0.461	**0.065**	0.699
	Eigenvector	0.369	0.887	0.320	0.677
Physical capital	Degree	0.265	–	0.883	0.299
	Betweenness	0.276	–	0.883	0.299
	Eigenvector	0.725	–	0.630	0.437
Financial capital	Degree	**0.052**	0.260	0.341	0.533
	Betweenness	**0.070**	0.322	0.640	0.262
	Eigenvector	0.774	0.850	0.884	0.979

### Climate regulation

The climate regulation network had fewer connections and was less dense than the timber production network (Fig. 4). The Biosphere Reserve administration in Schorfheide-Chorin (SCH Biosphere Reserve) took a central position in this network, scoring highest for degree centrality, betweenness centrality, and eigenvector centrality (Table S4). Here, we found differences in degree centrality for actor groups (*χ*^2^ = 11.20, d.f. = 6, *p* < 0.082, Table S5. Protected areas had the highest degree centrality (but no significant differences based on Dunn’s multiple comparison test (*p* < 0.01), Table S5. For eigenvector centrality, we found differences between actor groups when considering their influence concerning human capital for climate regulation specifically (*χ*^2^ = 7.700, d.f. = 3, *p* < 0.053; Table 2). The Biodiversity Exploratories and forest industry actors were the groups with the highest eigenvector centrality (but no significant differences based on Dunn’s multiple comparison test (*p* < 0.01), Table S7). Their influence can be executed via disseminating expert knowledge and using manual labour. This is referred to as *Pattern 2 governance of knowledge and labour for climate regulation*.

### Habitat creation and maintenance

The habitat creation and maintenance network included more actors (Fig. 5) than the climate regulation network. It was the only network in which five actors scored highest for degree centrality: an ornithologist (ALB Expert) and a forester (ALB Forester9) in the Schwäbische Alb, a forester (SCH Forester5) and the Biosphere Reserve administration (SCH Biosphere Reserve) in Schorfheide-Chorin, and the EU, which also scored highest for betweenness centrality. A local non-governmental organisation (NGO) in Schwäbische Alb scored highest for eigenvector centrality (ALB NGO) (Table S4). Among actor groups in this network, we found significant differences for degree centrality (*χ*^2^ = 13.003, d.f. = 6, *p* < 0.043) and betweenness centrality (*χ*^2^ = 14.502, d.f. = 6, *p* < 0.025). The protected areas actor group was most relevant in both (Dunn’s multiple comparison test (*p* < 0.01); Table S5). Similarly, we found differences for degree centrality (*χ*^2^ = 11.475, d.f. = 5, *p* < 0.043) and betweenness centrality (*χ*^2^ = 10.374, d.f. = 5, *p* < 0.065) among actor groups when considering their influence in the use of social capital to create and maintain habitat specifically (Table 2). Protected areas were the actor group with the highest degree centrality and betweenness centrality. Still, based on Dunn’s multiple comparison test (*p* < 0.01), Table S8), there were no significant differences between groups. Thus, protected areas were found to be well connected via social capital. This dual role of protected areas is described as *Pattern 3 governance for habitat management*.

### Non-material NCP

The non-material NCP network was more fragmented with more loosely connected actors than in the other NCP networks. Most interactions between actors were found in Schwäbische Alb (Fig. 6). Here, a local NGO (ALB NGO) scored highest for degree centrality, betweenness centrality, and eigenvector centrality, since it managed local infrastructure for recreational activities, such as hiking trails, and was connected to other actors by all four capital categories (Table S4). In contrast to the other networks, we neither found significant differences between actor groups for the co-production of non-material NCP (Table S5) nor between actor groups for the use of specific anthropogenic capitals (Table 2, Table S9). Hence, we could not derive a governance pattern for this NCP. Finding for few ties for non-material NCP and not being able to derive a governance pattern for these may be due the respondents’ background in forestry and conservation rather than tourism or local government.

## Discussion

Our analysis of data from 39 interviews about the co-production of multiple NCP in forests in three German sites uncovered three different actor constellations affecting anthropogenic capitals: (1) *governance of timber *via* financial flows*, (2) *governance of knowledge and labour for climate regulation*, and (3) *governance for habitat management*. Our findings provide useful insights into the trade-offs involved in the inclusive governance of diverse networks and can contribute to existing debates on NCP co-production (e.g. Bruley et al. 2021; Kachler et al. 2023; Giacomelli et al. 2024; Locatelli et al. 2024) and forest governance that seeks to achieve the sustainable supply of multiple forest NCP (e.g. Sheppard et al. 2020; Hernández-Morcillo et al. 2022; Mann et al. 2022; Lovrić et al. 2025).

### Actor contributions to co-producing forest NCP

The combined network of actor-to-actor relationships for four NCP, including material, regulating, and non-material NCP (Fig. 2), reflects the diverging and sometimes conflicting demands that forest management, in Germany and elsewhere, needs to account for and integrate (Borrass et al. 2017). Three main messages can be derived from the analysis of this network. First, regional context matters when discussing decision-making processes in forest management (Knoot and Rickenbach 2014). Biosphere reserves, for example, provide a setting in which the co-production of various NCP can be achieved (Palliwoda et al. 2021) and were established to enhance cooperation among actors (Stoll-Kleemann and O’Riordan 2018). Thus, they serve as a “designated coordinator” (Bodin 2017, 6). Our findings illustrate that biosphere reserves and their administrations fulfil a participatory and collaborative role in governing forest NCP co-production (Schultz et al. 2011), since they ranked high for centrality metrics in Schorfheide-Chorin (degree centrality) and Schwäbische Alb (eigenvector centrality). Whether this role applies for biosphere reserves regarding NCP co-production in other ecosystems needs further investigation.

Second, and as found for other social-ecological interactions (e.g. Barnes et al. 2017; Felipe-Lucia et al. 2021; Metzger et al. 2021), forest NCP co-production is governed by actor-to-actor relationships across multiple levels (e.g. Arts 2021; Hernández-Morcillo et al. 2022; Isaac et al. 2024b). These cross-level interactions tend to facilitate the coordination of actors at one, predominantly the lower, governance level (Bodin 2017). For example, without an explicit forest governance mandate, the EU indirectly coordinates forest actors at lower levels via various conservation policies, such as the Habitats Directive (92/43/EEC) (Elomina and Pülzl 2021). It is important to note that no organisation at the international level was mentioned by respondents concerning forest NCP co-production. For example, UNESCO only appeared in the declaration of names of the biosphere reserves, i.e. UNESCO Biosphere Reserve Schorfheide-Chorin.

Third, and advancing on Stoettner and Ní Dhubháin (2019) and Westin et al. (2023), who found forest owners’ values and degree of engagement within social networks influenced their management strategies, our findings for actor groups indicate that forest owners drive decisions in forest management by favouring specific anthropogenic capitals. Although we did not specifically test for different forms of power (see, e.g. Vallet et al. 2020), forest owners are able to exert power over other actors by influencing their choice of capitals. Like other powerful actors, they can frame management approaches and thus impact NCP provision (Morrison et al. 2019), for example, by choosing less invasive harvesting techniques despite higher costs to support the maintenance of habitat. Remarkably, and complementing the vital role of forest owners, we found the Biodiversity Exploratories, a scientific actor, to be the group with the highest betweenness centrality—underlining its role as relevant knowledge broker across the three sites. Thus, and on a positive note for science, research and science communication can contribute to facilitating collective learning via collaboration (Bodin 2017).

### Timber production

Timber production in the three sites appears to be strongly influenced by forestry contractors in Schorfheide-Chorin (highest degree centrality) and Schwäbische-Alb (highest eigenvector centrality) (Fig. 3). This finding is supported by Johansson et al. (2021), who describe contractor’s competence and resources, a human and a physical capital, as critical factors influencing their performance during timber harvest. Timber production and forests are also affected by certification schemes (Rametsteiner and Simula 2003). Thus, the central role of forest certification bodies (e.g. PEFC or FSC), is unsurprising since in 2019 75 to 83% of forests in Germany were certified (Umweltbundesamt 2020). Although forest owners’ and industry actors’ motivations differ when adopting certification schemes, they mainly aim to improve marketing opportunities and company appearance regarding sustainability (Zubizarreta et al. 2021). Despite prioritising material NCP, forest owners and industry actors in the three study sites perceived biodiversity loss as a significant risk to the economy (Peter et al. 2022). Timber production not only is a means to generate profits (Knoot and Rickenbach 2014), but to acquire financial capital, as illustrated by *pattern 1 governance of timber *via* financial flows*, needed for other anthropogenic capitals underpinning other less-income generating NCP (i.e. non-material and regulating forest NCP) (Lovrić et al. 2025). Thus, NCP co-production entails synergies between material and non-material NCP (Nicolás-Ruiz et al. 2023) and is based on complementarity between natural and anthropogenic capitals (Locatelli et al. 2024), as well as on complementarity between different forms of anthropogenic capitals.

### Climate regulation

Climate regulation within the sites depends on the biosphere reserve’s administration mediating role and the strict protection of forests in their core zones (Stoll-Kleemann and O’Riordan 2018). Protected forest has the highest carbon stocks in living biomass (Duncker et al. 2012). In contrast to this strict protection, the second pattern *governance of knowledge and labour for climate regulation* stresses that knowledge dissemination by expert organisations, such as the Biodiversity Exploratories, can contribute to improving climate regulation. This finding not only provides evidence that the *Biodiversity Exploratories* successfully fulfil their project aim (Fischer et al. 2010a), but contributes to what has been described as “cognitive co-production” by providing knowledge on the existence of climate regulation as an NCP (Giacomelli et al. 2024). Yet, when comparing to denser networks for timber production and habitat creation and maintenance, where co-production is mainly based on physical labour, the use of machinery and tools, or financial capital, the sharing of knowledge for climate regulation could still be enhanced through increasing collaborations in the three study sites. For example, for more transdisciplinary approaches, such as co-creation, co-production, or co-design of knowledge (Hakkarainen et al. 2021), involving scientists, forest actors, and policy makers could lead to better use of other anthropogenic capitals underpinning the capacity of forests to sequester carbon and regulate the micro-climate. Second and similar to the timber production network, forest industry actors, particularly contractors, appear to contribute to climate regulation through afforestation and reforestation practices (Jandl et al. 2007). These are labour-intensive and rely on suitable machinery, such as mechanised transplanters, and specific skills to operate these machines. Thus, our findings support the notion that NCP co-production largely depends on “people with ‘suitable’ identities and capabilities”, e.g. a specific skill set (Fischer and Eastwood 2016, 49).

### Habitat creation and maintenance

Collaboration among actors appears to be particularly relevant for habitat creation and maintenance in the three sites. Collaborative governance aims to balance diverging interests but does not necessarily entail solving social-ecological problems (Bodin 2017). Here, collaboration may contribute to creating and maintaining habitat but might fail to address the underlying causes of habitat loss, such as unsustainable forms of co-production, i.e. prioritising one NCP at the expense of others (Locatelli et al. 2024). Most often collaboration hinges on trust and information exchange between like-minded actors (Borg et al. 2015). For example, collaborative and participatory approaches in biosphere reserves were found to be more successful than strict top-down conservation measures (Stoll-Kleemann et al. 2010). Our findings further support this claim by illustrating how collaboration between local actors and an NGO in the Biosphere Schwäbische Alb can contribute to habitat conservation. Landscape maintenance, as performed by the NGO, is needed to keep the nutrient-poor grasslands open and free from reforestation as they are important habitats for endangered insects, such as specific butterfly (*Papilionoidea)* or grasshopper (*Caelifera*) species (Geschäftsstelle Biosphärengebiet Schwäbische Alb 2019; Geißler-Strobel and Herman 2023). Local NGOs are relevant actors in collaborative management approaches, for example regarding monitoring in Natura 2000 sites (Louette et al. 2015) or community-based management that fosters participation of locals in conservation measures (Friedman et al. 2020). National parks, in contrast, appear to engage less in collaborative processes as illustrated by our findings for the Hainich National Park. This may be due to the fact that fewer foresters manage sites in or adjacent to the park while all foresters in this study manage sites within the biosphere reserves.

Despite collaborative efforts at the case study level, our analysis indicated the need for complementary top-down approaches to maintain habitats. For example, conservation policies, such as the Habitats Directive (CD 92/43//EEC) and Birds Directive (CD 79/409/EEC), lead to the establishment of protected areas, such as Natura 2000 sites, whilst targeting collaboration among actors to manage these for the provision of multiple NCP (Schirpke et al. 2014; Pecurul-Botines et al. 2019). This has also been described as increasing “vertical fit” (Bodin 2017, 4–5). The dual nature of protected areas in fostering collaboration (e.g. actor-to-actor relationships) whilst enforcing top-down regulations (e.g. conservation laws) is highlighted in the third pattern *governance for habitat management* (Metzger et al. 2021). Protected areas fulfil these roles by acting as mediators between various governmental and civil society actors. As “designated coordinators” (Bodin 2017, 6), they bridge interests in the provision of forest NCP across sectors and governance levels (Keskitalo et al. 2014).

### Non-material NCP

Previous research has demonstrated how non-material NCP are provided and co-produced in protected areas and biosphere reserves in particular (Eastwood et al. 2016; Palliwoda et al. 2021). Good conservation status increases the potential for non-material NCP (Maes et al. 2012). For example, the Biosphere Reserve Schwäbische Alb is valued for its recreational opportunities by local and non-local visitors (Müller et al. 2019). They realise the potential supply of non-material NCP by engaging with nature, e.g. through recreational activities, such as hiking or cycling (Bieling 2014). Thus, non-material NCP depend on both their provision and realisation by their beneficiaries (Bruley et al. 2021).

Infrastructure, both recreational (e.g. picnic sites) and non-recreational (e.g. forest roads), supports the co-production of non-material NCP (Palliwoda et al. 2021). Good infrastructure facilitates easy access for visitors to natural areas and enhances opportunities to obtain non-material NCP (Schägner et al. 2016; Crouzat et al. 2022). Our findings suggest that collaboration among actors is useful for managing this infrastructure. Hiking trails in Schwäbische Alb, for example, are maintained by a local NGO and in close collaboration with the biosphere reserve administration, regional tourism organisations, conservation and forest authorities (Geschäftsstelle Biosphärengebiet Schwäbische Alb 2019). This contrasts with recent research highlighting that farmers in a Norwegian biosphere reserve contributed most to maintaining recreational paths (Barraclough et al. 2022). For the other two sites in this study and in contrast to the other NCP networks presented here, our focus on foresters and conservation managers may have led to few mentions of non-material NCP and recreational infrastructure. Future research should thus account for actor group diversity to broaden perspectives on the co-production of non-material NCP.

Like other research, our findings highlight the importance of social capital for co-producing non-material NCP (Kachler et al. 2023; Giacomelli et al. 2024), but further demonstrate that social capital mediates the management of physical capital (i.e. recreational infrastructure). More specifically, this supports the notion of biosphere reserves administrations as bridging actors who facilitate collaboration between state and non-state actors for non-material NCP provision (e.g. Schultz et al. 2011; Baird et al. 2018).

### Implications for research and decision-making

Understanding the drivers of NCP co-production is beneficial to land managers and decision-makers when managing social-ecological systems for sustainable forms of NCP co-production (Torralba et al. 2018; Locatelli et al. 2024). More specifically, NCP co-production depends on those actors holding agency over specific anthropogenic capitals (Felipe-Lucia et al. 2015; Bruley et al. 2021). We identified three patterns of actors driving the use of anthropogenic capitals in NCP co-production using SNA. These patterns have several implications for decision-making and future research.

First, land managers and owners depend on material NCP to generate financial capital to support other anthropogenic capitals and foster regulating and non-material NCP. For example, timber remains the major source of income in German forests despite the management aims of accounting for forest multifunctionality and meeting the societal demands for regulating and non-material NCP (Borrass et al. 2017; Hernández-Morcillo et al. 2022; Lovrić et al. 2025). This dependence is exhibited in two ways. For one, forest policy across governance levels in Germany favour timber production, for example, by setting rules for forestry associations that help small-scale forest owners to bring their timber to market (Schraml 2005; Isaac et al. 2024b). Moreover, our SNA showed a denser network for timber production than for the other NCP networks. This could imply a more distinct allocation of responsibilities among actors involved in the co-production of timber. To account for the multi-faceted NCP demands and to overcome the dependence on timber for financial income, policy-making needs to identify ways to support land managers and owners financially whilst providing regulation that incentivises less capital-intensive management approaches. Research can support this development by further investigating how synergies between different anthropogenic capitals can reduce financial dependencies.

Second, we showed that the knowledge sharing by scientific actors is valuable for climate regulation. While researchers have been criticised for falling short of communicating scientific findings and making them applicable to practice (Suldovsky et al. 2017), collaborative learning through participatory communication can be beneficial when addressing complex environmental problems (Bodin 2017). Researchers can make useful contributions by engaging more strongly with other actors, for example, through transdisciplinary approaches, to generate knowledge relevant for managing climate change mitigation and adaption actions (Chambers et al. 2021; Hakkarainen et al. 2021).

Third, actors collaborate via using anthropogenic capitals to create and maintain habitat but are coordinated by protected area staff. Protected area managers tend to take a mediating role if lacking formal power to enforce habitat conservation measures (Stoll-Kleemann et al. 2010). Nonetheless, power remains crucial when discussing the promotion of specific NCP over others (Juerges et al. 2020; Vallet et al. 2020). To support habitat creation and maintenance, research needs to further investigation is needed of how power dynamics and actor interests affect the prioritisation of specific anthropogenic capitals, which may lead to trade-offs between NCP. In turn, decision-makers need to be aware that favouring certain capitals, e.g. using heavy machinery to increase efficiency, may negatively impact regulating NCP.

Fourth, our findings showed that the co-production of non-material NCP was underrepresented when compared to the other NCP investigated in this study. While policy documents affecting our study sites require forests to be managed for the provision of multiple NCP (Isaac et al. 2024b), foresters and protected area representatives rarely appear to be involved in the co-production of non-material NCP, except for Schwäbische Alb where a local NGO and the biosphere reserve administration collaborate to maintain recreational infrastructure. Thus, future research needs to further investigate collaborations of forest actors, recreation and tourism focussed actors, and local governments to maintain recreational infrastructure. To support foresters in co-producing non-material NCP and meeting societal demands, forest policy across governance levels needs to establish incentive-based approaches, e.g. payments for ecosystem services (Hernández-Morcillo et al. 2022; Lovrić et al. 2025).

## Conclusion

We identified the most relevant actors in the management of different anthropogenic capitals involved in the co-production of forest NCP and derived governance patterns of these NCP. Our findings highlight that the co-production of material NCP across diverse governance networks is governed by a few actors and facilitated by financial capital flows. In contrast, the co-production of regulating NCP depends on collaboration between a broad range of actors and the coordination by protected areas. Furthermore, we found weak additional support for the governance of non-material NCP co-production related to collaboration between actors in managing infrastructure facilitating access to natural areas. We did, however, find that social capital mediates the management of physical capital in the co-production of non-material NCP. These findings contribute to a better understanding of the underlying actor relationships favouring the use of individual capitals in the co-production of NCP and could thus shape whether co-production processes can be sustainable.

## Supplementary Information

Below is the link to the electronic supplementary material.

Supplementary file1 (DOCX 480 KB)

## Data Availability

This work is based on data elaborated by the ESuDis project within the Biodiversity Exploratories program (DFG Priority Program 1374). The dataset (ID 31818) is available in the Biodiversity Exploratories Information System (10.48548/pubdata-217).

## Footnotes

1 United Nations Educational, Scientific and Cultural Organization.

## References

[CR1] Addinsoft. 2023. XLSTAT statistical and data analysis solution. New York.

[CR2] Aguilar Rodríguez, A., A. Langle-Flores, H. Romero-Uribe, J. Ros-Cuéllar, and J.J. Von Thaden. 2021. Multi-level social-ecological networks in a payments for ecosystem services programme in central Veracruz, Mexico. *Environmental Conservation* 48: 41–47. 10.1017/S0376892920000478.

[CR3] Alfasoft GmbH. 2023. NVIVO. Company Website.

[CR4] Allen, C.D., D.D. Breshears, and N.G. McDowell. 2015. On underestimation of global vulnerability to tree mortality and forest die-off from hotter drought in the Anthropocene. *Ecosphere* 6: 1–55. 10.1890/ES15-00203.1.

[CR5] Arts, B. 2021. Forest Governance: Hydra or Chloris?, 1st ed. Cambridge, United Kingdom; New York, USA; Port Melbourne, Australia; New Dehli, India; Visioncrest Commercial, Singapore: Cambridge University Press.

[CR6] Arts, B., and M. Buizer. 2009. Forests, discourses, institutions. *Forest Policy and Economics* 11: 340–347. 10.1016/j.forpol.2008.10.004.

[CR7] Baird, J., R. Plummer, L. Schultz, D. Armitage, and Örjan Bodin. 2018. Integrating Conservation and Sustainable Development Through Adaptive Co-management in UNESCO Biosphere Reserves. *Conservation and Society* 16. [Ashoka Trust for Research in Ecology and the Environment, Wolters Kluwer India Pvt. Ltd.]: 409–419. JSTOR.

[CR8] Barnes, M.L., Ö. Bodin, A.M. Guerrero, R.R.J. McAllister, S.M. Alexander, and G. Robins. 2017. The social structural foundations of adaptation and transformation in social—ecological systems. *Ecology and Society* 22: art16. 10.5751/ES-09769-220416.

[CR9] Barraclough, A.D., J. Cusens, and I.E. Måren. 2022. Mapping stakeholder networks for the co-production of multiple ecosystem services: A novel mixed-methods approach. *Ecosystem Services* 56: 101461. 10.1016/j.ecoser.2022.101461.

[CR10] Bastian, M., S. Heymann, and M. Jacomy. 2009. Gephi: An open source software for exploring and manipulating networks. Unpublished. 10.13140/2.1.1341.1520

[CR11] Bieling, C. 2014. Cultural ecosystem services as revealed through short stories from residents of the Swabian Alb (Germany). *Ecosystem Services* 8: 207–215. 10.1016/j.ecoser.2014.04.002.

[CR12] Biodiversity Exploratories Information System (BExIS). 2022. Borders of all three exploratory regions. *Borders of all three exploratory regions*.

[CR13] Biosphärengebiet Schwäbische Alb. 2023. Zahlen & Fakten.

[CR14] Bodin, Ö. 2017. Collaborative environmental governance: Achieving collective action in social-ecological systems. *Science* 357: eaan1114. 10.1126/science.aan1114. 28818915 10.1126/science.aan1114

[CR15] Bolte, A., C. Ammer, M. Löf, P. Madsen, G.-J. Nabuurs, P. Schall, P. Spathelf, and J. Rock. 2009. Adaptive forest management in central Europe: Climate change impacts, strategies and integrative concept. *Scandinavian Journal of Forest Research* 24: 473–482. 10.1080/02827580903418224.

[CR16] Borg, R., A. Toikka, and E. Primmer. 2015. Social capital and governance: A social network analysis of forest biodiversity collaboration in Central Finland. *Forest Policy and Economics* 50: 90–97. 10.1016/j.forpol.2014.06.008.

[CR17] Borgatti, S., M. Everett, J. Johnson, and F. Agneessens. 2022. In *Analyzing social networks using R*. First. Thousand Oaks: SAGE Publications.

[CR18] Borrass, L., D. Kleinschmit, and G. Winkel. 2017. The “German model” of integrative multifunctional forest management—analysing the emergence and political evolution of a forest management concept. *Forest Policy and Economics* 77: 16–23. 10.1016/j.forpol.2016.06.028.

[CR19] Brodribb, T.J., J. Powers, H. Cochard, and B. Choat. 2020. Hanging by a thread? Forests and drought. *Science* 368: 261–266. 10.1126/science.aat7631. 32299945 10.1126/science.aat7631

[CR20] Bruley, E., B. Locatelli, and S. Lavorel. 2021. Natures contributions to people: Coproducing quality of life from multifunctional landscapes. *Ecology and Society* 26: art12. 10.5751/ES-12031-260112.

[CR21] Chambers, J.M., C. Wyborn, M.E. Ryan, R.S. Reid, M. Riechers, A. Serban, N.J. Bennett, C. Cvitanovic, et al. 2021. Six modes of co-production for sustainability. *Nature Sustainability*. 10.1038/s41893-021-00755-x.

[CR22] Crouzat, E., A. De Frutos, V. Grescho, S. Carver, A. Büermann, C. Carvalho-Santos, R. Kraemer, S. Mayor, et al. 2022. Potential supply and actual use of cultural ecosystem services in mountain protected areas and their surroundings. *Ecosystem Services* 53: 101395. 10.1016/j.ecoser.2021.101395.

[CR23] Díaz, S., U. Pascual, M. Stenseke, B. Martín-López, R.T. Watson, Z. Molnár, R. Hill, K.M.A. Chan, et al. 2018. Assessing nature’s contributions to people. *Science* 359: 270–272. 10.1126/science.aap8826. 29348221 10.1126/science.aap8826

[CR24] Duncker, P.S., K. Raulund-Rasmussen, P. Gundersen, K. Katzensteiner, J. De Jong, H.P. Ravn, M. Smith, O. Eckmüllner, et al. 2012. How forest management affects ecosystem services, including timber production and economic return: Synergies and trade-offs. *Ecology and Society* 17: art50. 10.5751/ES-05066-170450.

[CR25] Eastwood, A., R. Brooker, R.J. Irvine, R.R.E. Artz, L.R. Norton, J.M. Bullock, L. Ross, D. Fielding, et al. 2016. Does nature conservation enhance ecosystem services delivery? *Ecosystem Services* 17: 152–162. 10.1016/j.ecoser.2015.12.001.

[CR26] Elomina, J., and H. Pülzl. 2021. How are forests framed? An analysis of EU forest policy. *Forest Policy and Economics* 127: 102448. 10.1016/j.forpol.2021.102448.

[CR27] Esri Deutschland GmbH. 2018. Bundesländergrenzen 2014 mit Einwohnerzahl.

[CR28] Felipe-Lucia, M.R., B. Martín-López, S. Lavorel, L. Berraquero-Díaz, J. Escalera-Reyes, and F.A. Comín. 2015. Ecosystem services flows: Why stakeholders’ power relationships matter. *Edited by Antoni Margalida. PLOS ONE* 10: e0132232. 10.1371/journal.pone.0132232. 10.1371/journal.pone.0132232 PMC4511803 26201000

[CR29] Felipe-Lucia, M.R., A.M. Guerrero, S.M. Alexander, J. Ashander, J.A. Baggio, M.L. Barnes, Ö. Bodin, A. Bonn, et al. 2021. Conceptualizing ecosystem services using social–ecological networks. *Trends in Ecology & Evolution*. 10.1016/j.tree.2021.11.012. 10.1016/j.tree.2021.11.012 34969536

[CR30] Felton, A., S. Belyazid, J. Eggers, E.-M. Nordström, and K. Öhman. 2023. Climate change adaptation and mitigation strategies for production forests: Trade-offs, synergies, and uncertainties in biodiversity and ecosystem services delivery in Northern Europe. *Ambio* 53: 1–16. 10.1007/s13280-023-01909-1. 37592197 10.1007/s13280-023-01909-1 PMC10692060

[CR31] Fischer, A., and A. Eastwood. 2016. Coproduction of ecosystem services as human–nature interactions—an analytical framework. *Land Use Policy* 52: 41–50. 10.1016/j.landusepol.2015.12.004.

[CR32] Fischer, M., E.K.V. Kalko, K.E. Linsenmair, S. Pfeiffer, D. Prati, E.-D. Schulze, and W.W. Weisser. 2010a. Exploratories for large-scale and long-term functional biodiversity research. In *Long-term ecological research*, ed. F. Müller, C. Baessler, H. Schubert, and S. Klotz, 429–443. Dordrecht: Springer Netherlands.

[CR33] Fischer, M., O. Bossdorf, S. Gockel, F. Hänsel, A. Hemp, D. Hessenmöller, G. Korte, J. Nieschulze, et al. 2010b. Implementing large-scale and long-term functional biodiversity research: The Biodiversity Exploratories. *Basic and Applied Ecology* 11: 473–485. 10.1016/j.baae.2010.07.009.

[CR34] Friedman, R.S., A.M. Guerrero, R.R.J. McAllister, J.R. Rhodes, T. Santika, S. Budiharta, T. Indrawan, J.A. Hutabarat, et al. 2020. Beyond the community in participatory forest management: A governance network perspective. *Land Use Policy* 97: 104738. 10.1016/j.landusepol.2020.104738.

[CR35] Garrido, P., M. Elbakidze, P. Angelstam, T. Plieninger, F. Pulido, and G. Moreno. 2017. Stakeholder perspectives of wood-pasture ecosystem services: A case study from Iberian Dehesas. *Land Use Policy* 60: 324–333. 10.1016/j.landusepol.2016.10.022.

[CR36] Geißler-Strobel, S., and G. Herman. 2023. *Erfolgskontrolle von Erst- pflegemaßnahmen des Projekts „Biotopverbund von Kalkmagerrasen im Raum Münsingen“ Tagfalter, Widderchen und Fingerkraut- Sandbiene*. Projekt: 22-048. Geschäftsstelle Biosphärengebiet Schwäbische Alb am Regierungspräsidium Tübingen.

[CR37] Geschäftsstelle Biosphärengebiet Schwäbische Alb. 2019. *Periodische Überprüfung des Biosphärenreservats Schwäbische Alb (2008–2019)*. Münsingen.

[CR38] Giacomelli, M., M. Sargolini, and M.R. Felipe-Lucia. 2024. Including the perspective of stakeholders in landscape planning through the Ecosystem Services co-production framework: An empirical exploration in Le Marche. *Italy. Regional Environmental Change* 24: 24. 10.1007/s10113-024-02184-w.

[CR39] Górriz-Mifsud, E., L. Secco, and E. Pisani. 2016. Exploring the interlinkages between governance and social capital: A dynamic model for forestry. *Forest Policy and Economics* 65: 25–36. 10.1016/j.forpol.2016.01.006.

[CR40] Górriz-Mifsud, E., L. Secco, R. Da Re, E. Pisani, and J.A. Bonet. 2017. Structural social capital and local-level forest governance: Do they inter-relate? A mushroom permit case in Catalonia. *Journal of Environmental Management* 188: 364–378. 10.1016/j.jenvman.2016.11.072. 28006745 10.1016/j.jenvman.2016.11.072

[CR41] Haddad, N.M., L.A. Brudvig, J. Clobert, K.F. Davies, A. Gonzalez, R.D. Holt, T.E. Lovejoy, J.O. Sexton, et al. 2015. Habitat fragmentation and its lasting impact on Earth’s ecosystems. *Science Advances* 1: e1500052. 10.1126/sciadv.1500052. 26601154 10.1126/sciadv.1500052 PMC4643828

[CR42] Hakkarainen, V., K. Mäkinen-Rostedt, A. Horcea-Milcu, D. D’Amato, J. Jämsä, and K. Soini. 2021. Transdisciplinary research in natural resources management: Towards an integrative and transformative use of co-concepts. *Sustainable Development*. 10.1002/sd.2276.

[CR43] Hannemann, R.A., and M. Riddle. 2011. 24 concepts and measures for basic network analysis. In *The SAGE handbook of social network analysis*, 1st ed., ed. J. Scott and P.J. Carrington, 340–369. London: SAGE Publications Ltd.

[CR44] Hernández-Morcillo, M., M. Torralba, T. Baiges, A. Bernasconi, G. Bottaro, S. Brogaard, F. Bussola, E. Díaz-Varela, et al. 2022. Scanning the solutions for the sustainable supply of forest ecosystem services in Europe. *Sustainability Science*. 10.1007/s11625-022-01111-4. 10.1007/s11625-022-01111-4 PMC8939503 35340343

[CR46] Isaac, R., J. Kachler, B. Martín-López, and M. Felipe-Lucia. 2024a. Fieldwork protocol: Effects of land management on the Supply and Distribution of ecosystem services (ESuDis). Application/pdf (version 1). [object Object]: 3491757 b. 10.48548/PUBDATA-218.

[CR45] Isaac, R., J. Hofmann, J. Koegst, C. Schleyer, and B. Martín-López. 2024b. Governing anthropogenic assets for nature’s contributions to people in forests: A policy document analysis. *Environmental Science & Policy* 152: 103657. 10.1016/j.envsci.2023.103657.

[CR47] Jandl, R., M. Lindner, L. Vesterdal, B. Bauwens, R. Baritz, F. Hagedorn, D.W. Johnson, K. Minkkinen, et al. 2007. How strongly can forest management influence soil carbon sequestration? *Geoderma* 137: 253–268. 10.1016/j.geoderma.2006.09.003.

[CR48] Jericó-Daminello, C., B. Schröter, M. Mancilla Garcia, and C. Albert. 2021. Exploring perceptions of stakeholder roles in ecosystem services coproduction. *Ecosystem Services* 51: 101353. 10.1016/j.ecoser.2021.101353.

[CR49] Johansson, M., E. Erlandsson, T. Kronholm, and O. Lindroos. 2021. Key drivers and obstacles for performance among forest harvesting service contractors—a qualitative case study from Sweden. *Scandinavian Journal of Forest Research* 36: 598–613. 10.1080/02827581.2021.1981431.

[CR50] Juerges, N., B. Arts, M. Masiero, E.Z. Başkent, J.G. Borges, Y. Brodrechtova, V. Brukas, M.J. Canadas, et al. 2020. Integrating ecosystem services in power analysis in forest governance: A comparison across nine European countries. *Forest Policy and Economics* 121: 102317. 10.1016/j.forpol.2020.102317.

[CR51] Kachler, J., R. Isaac, B. Martín-López, A. Bonn, and M.R. Felipe-Lucia. 2023. Co-production of nature’s contributions to people: What evidence is out there? *People and Nature*. 10.1002/pan3.10493.

[CR52] Kachler, J., M.R. Felipe-Lucia, R. Isaac, A. Bonn, and B. Martín-López. 2024. Intrinsic, instrumental and relational values behind nature’s contributions to people preferences of nature visitors in Germany. *Ecosystems and People* 20: 2342361. 10.1080/26395916.2024.2342361.

[CR53] Keskitalo, E.C.H. 2013. Understanding adaptive capacity in forest governance: Editorial. *Ecology and Society* 18: art45. 10.5751/ES-05924-180445.

[CR54] Keskitalo, E.C.H., J. Baird, E. Laszlo Ambjörnsson, and R. Plummer. 2014. Social network analysis of multi-level linkages: A Swedish case study on northern forest-based sectors. *Ambio* 43: 745–758. 10.1007/s13280-014-0492-0. 24570210 10.1007/s13280-014-0492-0 PMC4165835

[CR55] Knoot, T.G., and M. Rickenbach. 2014. Forester networks: The intersection of private lands policy and collaborative capacity. *Land Use Policy* 38: 388–396. 10.1016/j.landusepol.2013.11.025.

[CR56] Korhonen, K., T. Hujala, and M. Kurttila. 2012. Reaching forest owners through their social networks in timber sales. *Scandinavian Journal of Forest Research* 27: 88–99. 10.1080/02827581.2011.631935.

[CR57] Landesamt für Umwelt Brandenburg. 2023. Biosphärenreservat Schorfheide-Chorin.

[CR58] Lavorel, S., B. Locatelli, M.J. Colloff, and E. Bruley. 2020. Co-producing ecosystem services for adapting to climate change. *Philosophical Transactions of the Royal Society B: Biological Sciences* 375: 20190119. 10.1098/rstb.2019.0119. 10.1098/rstb.2019.0119 PMC7017776 31983325

[CR59] Locatelli, B., E.M. Bennett, M.J. Colloff, M.R. Felipe-Lucia, R. Gorddard, I. Palomo, and S. Lavorel. 2024. People working with nature: A theoretical perspective on the co-production of nature’s contributions to people. *Ecosystems and People* 20: 2359061. 10.1080/26395916.2024.2359061.

[CR60] Louette, G., D. Adriaens, D. Paelinckx, and M. Hoffmann. 2015. Implementing the habitats directive: How science can support decision making. *Journal for Nature Conservation* 23: 27–34. 10.1016/j.jnc.2014.12.002.

[CR61] Lovrić, M., M. Torralba, F. Orsi, D. Pettenella, C. Mann, D. Geneletti, T. Plieninger, E. Primmer, et al. 2025. Mind the income gap: Income from wood production exceed income from providing diverse ecosystem services from Europe’s forests. *Ecosystem Services* 71: 101689. 10.1016/j.ecoser.2024.101689.

[CR62] Maes, J., M.L. Paracchini, G. Zulian, M.B. Dunbar, and R. Alkemade. 2012. Synergies and trade-offs between ecosystem service supply, biodiversity, and habitat conservation status in Europe. *Biological Conservation* 155: 1–12. 10.1016/j.biocon.2012.06.016.

[CR63] Mann, C., L. Loft, M. Hernández-Morcillo, E. Primmer, F. Bussola, E. Falco, D. Geneletti, E. Dobrowolska, et al. 2022. Governance Innovations for forest ecosystem service provision—insights from an EU-wide survey. *Environmental Science & Policy* 132: 282–295. 10.1016/j.envsci.2022.02.032. 35663433 10.1016/j.envsci.2022.02.032 PMC8996823

[CR64] Marin, A., and B. Wellman. 2011. 2 social network analysis: An introduction. In *The SAGE handbook of social network analysis*, 1st ed., ed. J. Scott and P. Carrington, 11–25. London: SAGE Publications Ltd.

[CR66] Metzger, J.P., P. Fidelman, C. Sattler, B. Schröter, M. Maron, F. Eigenbrod, M. Fortin, C. Hohlenwerger, et al. 2021. Connecting governance interventions to ecosystem services provision: A social‐ecological network approach. Edited by Unai Pascual. *People and Nature* 3: 266–280. 10.1002/pan3.10172.

[CR67] Morrison, T.H., W.N. Adger, K. Brown, M.C. Lemos, D. Huitema, J. Phelps, L. Evans, P. Cohen, et al. 2019. The black box of power in polycentric environmental governance. *Global Environmental Change* 57: 101934. 10.1016/j.gloenvcha.2019.101934.

[CR68] Müller, S.M., J. Peisker, C. Bieling, K. Linnemann, K. Reidl, and K. Schmieder. 2019. The importance of cultural ecosystem services and biodiversity for landscape visitors in the biosphere reserve Swabian Alb (Germany). *Sustainability* 11: 2650. 10.3390/su11092650.

[CR69] Nationalpark Hainich. 2023. Nationalpark Hainich. Administration.

[CR70] Nicolás-Ruiz, N., C. Quintas-Soriano, M.L. Suárez, and M. Rosario Vidal-Abarca. 2023. Co-production of nature’s contributions to people in dry rivers: A case study in Murcia Spain. *Ecosystems and People* 19: 2288953. 10.1080/26395916.2023.2288953.

[CR71] Oteros-Rozas, E., B. Martín-López, J.A. González, T. Plieninger, C.A. López, and C. Montes. 2014. Socio-cultural valuation of ecosystem services in a transhumance social-ecological network. *Regional Environmental Change* 14: 1269–1289. 10.1007/s10113-013-0571-y.

[CR72] Palliwoda, J., J. Fischer, M.R. Felipe-Lucia, I. Palomo, R. Neugarten, A. Büermann, M.F. Price, M. Torralba, et al. 2021. Ecosystem service coproduction across the zones of biosphere reserves in Europe. *Ecosystems and People* 17: 491–506. 10.1080/26395916.2021.1968501.

[CR73] Palomo, I., M.R. Felipe-Lucia, E.M. Bennett, B. Martín-López, and U. Pascual. 2016. Disentangling the pathways and effects of ecosystem service co-production. *Advances in Ecological Research* 54: 245–283. 10.1016/bs.aecr.2015.09.003.

[CR74] Pecurul-Botines, M., M. Di Gregorio, and J. Paavola. 2019. Multi-level processes and the institutionalization of forest conservation discourses: Insights from Natura 2000. *Forest Policy and Economics* 105: 136–145. 10.1016/j.forpol.2019.05.027.

[CR75] Peter, S., G. Le Provost, M. Mehring, T. Müller, and P. Manning. 2022. Cultural worldviews consistently explain bundles of ecosystem service prioritisation across rural Germany. *People and Nature* 4: 218–230. 10.1002/pan3.10277.

[CR76] Primmer, E., L. Varumo, T. Krause, F. Orsi, D. Geneletti, S. Brogaard, E. Aukes, M. Ciolli, et al. 2021. Mapping Europe’s institutional landscape for forest ecosystem service provision, innovations and governance. *Ecosystem Services* 47: 101225. 10.1016/j.ecoser.2020.101225.

[CR77] Pukkala, T. 2016. Which type of forest management provides most ecosystem services? *Forest Ecosystems* 3: 9. 10.1186/s40663-016-0068-5.

[CR78] Rametsteiner, E., and M. Simula. 2003. Forest certification—an instrument to promote sustainable forest management? *Journal of Environmental Management* 67: 87–98. 10.1016/S0301-4797(02)00191-3. 12659807 10.1016/s0301-4797(02)00191-3

[CR79] Reyers, B., R. Biggs, G.S. Cumming, T. Elmqvist, A.P. Hejnowicz, and S. Polasky. 2013. Getting the measure of ecosystem services: A social–ecological approach. *Frontiers in Ecology and the Environment* 11: 268–273. 10.1890/120144.

[CR80] Rieb, J.T., B.E. Robinson, and E.M. Bennett. 2023. Substitutability of natural and human capitals: Lessons from a simple exploratory model. *Ecosystems and People* 19: 2281483. 10.1080/26395916.2023.2281483.

[CR81] Schägner, J.P., L. Brander, J. Maes, M.L. Paracchini, and V. Hartje. 2016. Mapping recreational visits and values of European National Parks by combining statistical modelling and unit value transfer. *Journal for Nature Conservation* 31: 71–84. 10.1016/j.jnc.2016.03.001.

[CR82] Schirpke, U., R. Scolozzi, C. De Marco, and U. Tappeiner. 2014. Mapping beneficiaries of ecosystem services flows from Natura 2000 sites. *Ecosystem Services* 9: 170–179. 10.1016/j.ecoser.2014.06.003.

[CR83] Schraml, U. 2005. Between legitimacy and efficiency: The development of forestry associations in Germany. *Small-Scale Forest Economics, Management and Policy* 4: 251–267. 10.1007/s11842-005-0016-7.

[CR84] Schultz, L., A. Duit, and C. Folke. 2011. Participation, adaptive co-management, and management performance in the world network of biosphere reserves. *World Development* 39: 662–671. 10.1016/j.worlddev.2010.09.014.

[CR85] Schulz, T., E. Lieberherr, and A. Zabel. 2018. Network governance in national Swiss forest policy: Balancing effectiveness and legitimacy. *Forest Policy and Economics* 89: 42–53. 10.1016/j.forpol.2016.10.011.

[CR86] Sheppard, J.P., J. Chamberlain, D. Agúndez, P. Bhattacharya, P.W. Chirwa, A. Gontcharov, W.C.J. Sagona, H. Shen, et al. 2020. Sustainable forest management beyond the timber-oriented status quo: Transitioning to co-production of timber and non-wood forest products—a global perspective. *Current Forestry Reports* 6: 26–40. 10.1007/s40725-019-00107-1.

[CR87] Smith, M., A. Ceni, N. Milic-Frayling, B. Shneiderman, E. Mendes Rodrigues, J. Leskovec, and C. Dunne. 2010. NodeXL: A free and open network overview, discovery and exploration add-in for Excel 2007/2010/2013/2016. Social Media Research Foundation.

[CR88] Sotirov, M., and S. Storch. 2018. Resilience through policy integration in Europe? Domestic forest policy changes as response to absorb pressure to integrate biodiversity conservation, bioenergy use and climate protection in France, Germany, the Netherlands and Sweden. *Land Use Policy* 79: 977–989. 10.1016/j.landusepol.2017.04.034.

[CR89] Spangenberg, J.H., C. Görg, D.T. Truong, V. Tekken, J.V. Bustamante, and J. Settele. 2014. Provision of ecosystem services is determined by human agency, not ecosystem functions. Four case studies. *International Journal of Biodiversity Science, Ecosystem Services & Management* 10: 40–53. 10.1080/21513732.2014.884166.

[CR90] Stoettner, E.M., and Á. Ní Dhubháin. 2019. The social networks of Irish private forest owners: An exploratory study. *Forest Policy and Economics* 99: 68–76. 10.1016/j.forpol.2017.09.008.

[CR91] Stoll-Kleemann, S., A.C. De La Vega-Leinert, and L. Schultz. 2010. The role of community participation in the effectiveness of UNESCO Biosphere Reserve management: Evidence and reflections from two parallel global surveys. *Environmental Conservation* 37: 227–238. 10.1017/S037689291000038X.

[CR92] Stoll-Kleemann, S., and T. O’Riordan. 2018. Biosphere Reserves in the Anthropocene. 347–353. 10.1016/B978-0-12-809665-9.09828.

[CR93] Suldovsky, B., B. McGreavy, and L. Lindenfeld. 2017. Science communication and stakeholder expertise: Insights from sustainability science. *Environmental Communication* 11: 587–592. 10.1080/17524032.2017.1308408.

[CR94] Torralba, M., E. Oteros-Rozas, G. Moreno, and T. Plieninger. 2018. Exploring the role of management in the coproduction of ecosystem services from Spanish wooded rangelands. *Rangeland Ecology & Management* 71: 549–559. 10.1016/j.rama.2017.09.001.

[CR95] Umweltbundesamt. 2020. Umweltschutz, Wald und nachhaltige Holznutzung in Deutschland. Umweltbundesamt.

[CR96] UNESCO. 2023. Ancient and Primeval Beech Forests of the Carpathians and Other Regions of Europe.

[CR97] Vallet, A., B. Locatelli, C. Barnaud, D. Makowski, Y. Quispe Conde, and H. Levrel. 2020. Power asymmetries in social networks of ecosystem services governance. *Environmental Science & Policy* 114: 329–340. 10.1016/j.envsci.2020.08.020.

[CR98] van der Hulst, R.C. 2011. 18 terrorist networks: The threat of connectivity. In *The SAGE handbook of social network analysis*, ed. J. Scott and P.J. Carrington, 256–270. London: SAGE Publications Ltd.

[CR101] Westin, K., A. Bolte, E. Haeler, E. Haltia, R. Jandl, A. Juutinen, K. Kuhlmey, G. Lidestav, et al. 2023. Forest values and application of different management activities among small-scale forest owners in five EU countries. *Forest Policy and Economics* 146: 102881. 10.1016/j.forpol.2022.102881.

[CR99] Yibarbuk, D., P.J. Whitehead, J. Russell-Smith, D. Jackson, C. Godjuwa, A. Fisher, P. Cooke, D. Choquenot, et al. 2001. Fire ecology and Aboriginal land management in central Arnhem Land, northern Australia: A tradition of ecosystem management. *Journal of Biogeography* 28: 325–343. 10.1046/j.1365-2699.2001.00555.x.

[CR100] Zubizarreta, M., G. Arana-Landín, and J. Cuadrado. 2021. Forest certification in Spain: Analysis of certification drivers. *Journal of Cleaner Production* 294: 126267. 10.1016/j.jclepro.2021.126267.

