# Supplementary material for: The forest beyond the trees: A network perspective on governing co-production of nature’s contributions to people
Source: Ambio. 2025 Apr 26;54(11):1835–51. doi: 10.1007/s13280-025-02187-9 (PMC12480197; doi:10.1007/s13280-025-02187-9)
Supplement: Supplementary file 1 — Supplementary file1 (DOCX 480 KB) [file 13280_2025_2187_MOESM1_ESM.pdf]

Ambio

Supplementary Information
This supplementary information has not been peer reviewed.

Title: **The forest beyond the trees: a network perspective to identify archetypes of governing nature's contributions to people co-production**

S1 Table. Defining anthropogenic capitals that underpin nature's contributions to people co-production. Adapted from Isaac et al. (2022) and Isaac et al. (2024) as well as based on Palomo et al. (2016).

Type of anthropogenic capital	*Definition*	*Forest related examples*
Human capital	Includes people's capabilities such as health, knowledge, skills, motivation, and labour that contribute to the provision of NCP.	Forest management, forestry specific knowledge, habitat specific knowledge.
Social capital	Any form of interaction between people through networks, trust or within or between institutions that supports the provision of NCP.	Forestry associations that organise forest management and timber harvest. Formal institutions, such as laws, that regulate the use and management of forests and their products.
Physical capital	Manufactured or technological capitals that are used to provide NCP. Infrastructure and built capital that is used to access NCP.	Machinery and tools used in forestry such as chainsaws, harvesters, trucks, or fences. Logging roads or footpaths.

Financial capital	Any form of financial stream in the form of savings, credits, subsidies, direct payments or revenue that enables the use of other anthropogenic capitals and thereby underpins the provision of NCP.	Governmental direct payments to leave deadwood in forests. Bank loans to buy machinery.

References

Isaac, R., Hofmann, J., Koegst, J., Schleyer, C., Martín-López, B., 2024. Governing anthropogenic assets for nature's contributions to people in forests: a policy document analysis. Environmental Science & Policy 152, 103657. https://doi.org/10.1016/j.envsci.2023.103657

Isaac, R., Kachler, J., Winkler, K.J., Albrecht, E., Felipe-Lucia, M.R., Martín-López, B., 2022. Governance to manage the complexity of nature's contributions to people co-production, in: Advances in Ecological Research. Elsevier, p. S0065250422000095. https://doi.org/10.1016/bs.aecr.2022.04.009

Palomo, I., Felipe-Lucia, M.R., Bennett, E.M., Martín-López, B., Pascual, U., 2016. Disentangling the Pathways and Effects of Ecosystem Service Co-Production, in: Advances in Ecological Research. Elsevier, pp. 245–283. https://doi.org/10.1016/bs.aecr.2015.09.003

S2 Table. Coding set for the interview transcripts to elicit actor to actor relationships in the co-production of nature's contributions to people in forests. Based on Isaac et al. (2022) S4.

Category	*Code*	*Description*	*Definition (if applicable)*	*Selection*
1) general info	interview ID	identification number of the interview		number (continuous)
	coder	person coding the policy document		RI, JK, BML
	exploratory	name of the exploratory		SCH; HAI; ALB
	verbatim	verbatim describing the actor-to-actor relationship, the NCP,		text

		and anthropogenic capital	
2) actor to actor relationship	actor A	anonymised acronym of actor A	text
	actor group A	actor group to which this actor belongs	text
	level actor A	level at which actor A operates	SCH; HAI; ALB, Brandenburg; Thuringia; Baden-Württemberg; Germany; European Union
	actor B	anonymised acronym of actor B	text
	actor group B	actor group to which this actor belongs	text
	level actor B	level at which actor B operates	SCH; HAI; ALB, Brandenburg; Thuringia; Baden-Württemberg; Germany; European Union
3) NCP group	NCP group	category to which the co-produced NCP belongs	material; regulating; non-material
	focal forest NCP	sub-category to which the NCP belongs to in case that it is a material (defined in previous column) (see sub-categories on the right)	timber production; carbon sequestration; micro-climate; pollination; natural pest control; habitat management; physical activities; observation and enjoyment of plants; observation and enjoyment of animals; landscape enjoyment
	NCP (in words)	describe NCP in words	text
	NCP group	grouped individual NCP	timber production; climate regulation; habitat creation and maintenance; non-material NCP

4) anthropogenic capitals	human capital category	category to which human capital belongs to (if applicable)	Human capital refers to people's capabilities including health, informal knowledge, formal knowledge, skills, motivation, and labour that can yield a flow of NCP (Goodwin, 2003; Palomo et al., 2016).	labour; knowledge; skills; other HC
	human capital description	describe human capital indicator in words		text
	social capital category	category to which social capital belongs to (if applicable)	Social capital includes all intangible assets associated with interactions between people including formal and informal networks, trust, and formal and informal institutions required for enhancing the flow of NCP. (Palomo et al., 2016)	networks; informal institutions; other SC
	social capital description	describe social capital indicator in words		text
	physical capital category	category to which physical capital belongs to (if applicable)	Physical capital refers to technological or manufactured assets that contribute to the flow of NCP (Goodwin, 2003; Palomo et al., 2016; Pretty, 2003).	tools and machinery; infrastructure and built capital; other PC

	physical capital description	describe physical capital indicator in words		text
	financial capital	if co-production involves financial capital	Financial capital - virtual mechanisms in the form of savings, credits, and other monetary forms used for trading, maintaining, or enhancing natural, human, social or physical capitals that contribute to the flow of NCP. (Palomo et al., 2016)	direct payments; subsidies; other FC
	financial capital description	describe financial capital indicator in words		text
5) general comments				text

References

Goodwin, N.R., 2003. Five Kinds of Capital: Useful Concepts for Sustainable Development.

Isaac, R., Kachler, J., Winkler, K.J., Albrecht, E., Felipe-Lucia, M.R., Martín-López, B., 2022. Governance to manage the complexity of nature's contributions to people co-production, in: Advances in Ecological Research. Elsevier, p. S0065250422000095. https://doi.org/10.1016/bs.aecr.2022.04.009

Palomo, I., Felipe-Lucia, M.R., Bennett, E.M., Martín-López, B., Pascual, U., 2016. Disentangling the Pathways and Effects of Ecosystem Service Co-Production, in: Advances in Ecological Research. Elsevier, pp. 245–283. https://doi.org/10.1016/bs.aecr.2015.09.003

Pretty, J., 2003. Social Capital and the Collective Management of Resources. Science 302, 1912–1914. https://doi.org/10.1126/science.1090847

S3 Text. Description of Social Network Analysis (SNA) and the calculated centrality metrics.

Social networks are formed by actors via one or multiple interactions (Marin and Wellman, 2011). Actors within social networks are referred to as nodes, their connections are known as ties. Attributes are the characteristics describing nodes or ties. For example, for nodes, these might be an actor's profession, age, or gender (Borgatti et al., 2022); or for ties, the quality, speed, or some other property beyond the interaction that creates the tie. Ties can be directed, indicating, for example, the flow of information from one node to another but not vice-versa, or undirected, as in our study (Marin and Wellman, 2011). In this study, we treat the actor's group membership as their primary attribute of interest.

In SNA, centrality measures provide insights into how individual nodes might affect flows through the broader network (Hannemann and Riddle, 2011). We focus on three measures of centrality. First, degree centrality measures "the number of ties of a given type that a node has" (Borgatti et al., 2022, p. 171). It is the simplest centrality measure and solely describes local connectivity. Second, betweenness centrality measures how often a node is on the shortest path between two otherwise detached nodes (Borgatti et al., 2022). The higher a node's betweenness centrality, the more significant its role in connecting other groups of nodes and potentially the higher its level of control over whole-network dynamics (Hannemann and Riddle, 2011). Thus, nodes with high betweenness centrality act as gatekeepers (Borgatti et al., 2022); they may, for example, have low degree centrality while providing a critical connection between two nodes that would otherwise fall in separate networks. Third, eigenvector centrality measures how often a node is connected to other well-connected nodes and can thus be interpreted "as a measure of popularity in the sense that a node with high eigenvector centrality is connected to nodes that are themselves well connected" (Borgatti et al.,

2022, p. 173). These three centrality measures typically exhibit considerable redundancy; we measured all three because each illuminates a slightly different aspect of a node's contribution to network structure and potential function.

References

Borgatti, S., Everett, M., Johnson, J., Agneessens, F., 2022. Analyzing social networks using R, First. ed. SAGE Publications, Thousand Oaks.

Hannemann, R.A., Riddle, M., 2011. 24 Concepts and Measures for Basic Network Analysis, in: Scott, J., Carrington, P.J. (Eds.), The SAGE Handbook of Social Network Analysis. SAGE Publications Ltd, 1 Oliver's Yard, 55 City Road, London EC1Y 1SP United Kingdom, pp. 340–369.

Marin, A., Wellman, B., 2011. 2 Social Network Analysis: An Introduction, in: Scott, J., Carrington, P. (Eds.), The SAGE Handbook of Social Network Analysis. SAGE Publications Ltd, 1 Oliver's Yard, 55 City Road, London EC1Y 1SP United Kingdom, pp. 11–25.

S4 Table. Centrality metrics (degree, betweenness, eigenvector) for each individual actor and for all nature's contributions to people in this study (timber, climate regulation, habitat creation and maintenance, non-material NCP) and their combination. Bold numbers highlight those with the highest score for each category.

		All nature's contributions to people			Timber			Climate regulation			Habitat creation and maintenance			Non-material nature's contributions to people		
**Actor**	**Actor Group**	**Degree**	**Betweennes**	**Eigenvector**	**Degree**	**Betweenness**	**Eigenvector**	**Degree**	**Betweennes**	**Eigenvector**	**Degree**	**Betweennes**	**Eigenvector**	**Degree**	**Betweenness**	**Eigenvector**
ALBagriculture	Societalactors	2	0	0.052702	-	-	-	-	-	-	2	0	0.154561	-	-	-
ALBbiosphereresrve	Protectedareas	**11**	**217.166556**	**0.276418**	-	-	-	**3**	9	0	**6**	159.762338	**0.283819**	**5**	13.852381	**0.368481**
ALBcontractors	Forestindustry	**9**	126.41962	0.221016	**9**	193.157143	**0.304643**	-	-	-	1	0	0.076488	1	0	0.079485
ALBeducation	Societalactors	1	0	0.019246	1	0	0.025619	-	-	-	-	-	-	-	-	-
ALBenvironmentalNGO	Societalactors	**11**	**178.540656**	**0.245341**	-	-	-	-	-	-	**6**	77.950794	**0.342105**	**8**	**59.47381**	**0.443047**
ALBexpert	Societalactors	7	104.725711	0.190999	-	-	-	-	-	-	**7**	203.702814	**0.321786**	2	5.761905	0.115692
ALBforestauthority	Government	8	291.576782	0.196662	**5**	21.848016	0.196956	-	-	-	**4**	112.055628	0.194738	**5**	**35**	**0.290758**
ALBforester1	Foresters	7	116.945791	0.144005	**5**	94.687302	0.155966	1	0	0	-	-	-	**3**	2.785714	0.238901
ALBforester10	Foresters	8	**17.054152**	**0.236662**	3	7.018254	0.132374	-	-	-	1	0	0.073436	**6**	**37.383333**	**0.348786**
ALBforester11	Foresters	2	0.5	0.032973	-	-	-	-	-	-	1	0	0.073436	2	0.75	0.06458
ALBforester12	Foresters	**9**	144.280918	0.207308	**7**	362.744444	**0.265604**	-	-	-	**4**	48.102525	0.208995	-	-	-
ALBforester2	Foresters	**11**	**403.920297**	**0.263777**	**6**	54.328175	0.220928	-	-	-	**6**	**511.352525**	**0.304702**	-	-	-
ALBforester3	Foresters	**11**	333.806282	0.217564	**6**	336.689683	**0.227171**	-	-	-	1	0	0.073436	**4**	**18.452381**	0.260819
ALBforester5	Foresters	7	111.324008	0.157413	4	13.134921	0.173488	-	-	-	**4**	130	0.069742	2	0.983333	0.152973
ALBforester6	Foresters	1	0	0.020213	-	-	-	-	-	-	1	0	0.015916	-	-	-
ALBforester7	Foresters	7	254.350097	0.153223	**6**	369.84127	0.2232	-	-	-	1	0	0.045853	-	-	-
ALBforester8	Foresters	3	0.780154	0.052602	3	4.457937	0.095395	-	-	-	1	0	0.01864	-	-	-
ALBforester9	Foresters	8	89.652089	0.165078	2	2.324603	0.082586	**2**	5	0	**7**	179.700577	**0.335159**	1	0	0
ALBforesters	Foresters	8	139.91541	0.149878	**6**	107.139683	0.137006	**2**	8	0	2	6	0.034556	-	-	-
ALBforestindustry	Forestindustry	6	41.952184	0.156058	**6**	59.189683	0.219868	-	-	-	-	-	-	-	-	-
ALBforestlandusers	Forestindustry	1	0	0.035495	-	-	-	-	-	-	1	0	0.064771	-	-	-
ALBforestowners	Forestowners	5	256.079025	0.123475	3	4.52381	0.10682	1	0	0.001836	**3**	118.411111	0.12959	-	-	-
ALBgovernment	Government	**13**	**502.245692**	**0.326008**	**6**	67.37381	**0.248303**	**3**	5	0	**3**	5.466667	0.207745	**6**	**32.042857**	**0.331743**
ALBsociety	Societalactors	1	0	0.027938	-	-	-	-	-	-	-	-	-	1	0	0.059439
ALBtourism	Societalactors	4	10.021195	0.123788	-	-	-	-	-	-	-	-	-	**4**	6.566667	0.273465
ALBtourists	Societalactors	3	16.192922	0.065781	-	-	-	-	-	-	-	-	-	**3**	11.471429	0.167692
ALBtrainees	Foresters	2	0.738095	0.052364	2	1.208333	0.070477	-	-	-	-	-	-	-	-	-
ALBworkers	Forestindustry	7	45.055961	0.199389	**7**	124.790873	**0.266252**	-	-	-	1	0	0.015916	1	0	0.079485
AUTforestagency	Government	1	0	0.027938	1	0	0.042481	-	-	-	-	-	-	-	-	-
BBscience&education	Societalactors	1	0	0.006939	-	-	-	1	0	0.079828	-	-	-	-	-	-
BBstate	Government	**10**	130.150486	0.064806	**7**	77.095737	0.064214	**3**	3	**0.338058**	**5**	143.734199	0.10258	1	0	0
BBstateforest	Government	8	93.134024	0.053596	**5**	163.295771	0.056388	**2**	29	0.192238	**4**	89.12619	0.094078	-	-	-
BWforestauthority	Government	1	0	0.018492	1	0	0.029165	-	-	-	-	-	-	-	-	-
BWscience&education	Societalactors	5	21.783844	0.134882	4	23.199206	0.170291	1	0	0	**3**	18.115079	0.168625	1	0	0.066261
BWstate	Government	**9**	148.015968	0.233955	2	10.97619	0.069643	**2**	0	0	**5**	244.596104	0.239211	**4**	11.47619	0.228167
BWstateforest	Government	5	15.550189	0.106533	4	19.401587	0.149933	-	-	-	**3**	101	0.081678	-	-	-
EU	Government	**11**	**1303.17919**	0.156538	3	158.92186	0.035334	1	0	0.177879	**7**	**673.827201**	0.200917	1	0	0.075605
GERenvironmentalNGO	Societalactors	3	103.522827	0.056768	-	-	-	-	-	-	**3**	145.827994	0.165843	2	2	0
GERexploratories	BiodiversityExploratori	5	483.63485	0.051136	1	0	0.010507	**2**	**225**	**0.197713**	**3**	138.617893	0.058766	1	0	0.066261
GERforestcertification	Societalactors	6	468.909648	0.090516	**6**	**1207.167502**	0.211266	-	-	-	-	-	-	-	-	-
GERforestryNGO	Societalactors	3	63.988286	0.016436	3	97.558983	0.05084	-	-	-	1	0	0.04166	-	-	-
GERscience	Societalactors	1	0	0.013566	-	-	-	1	0	0.177879	-	-	-	-	-	-
GERsociety	Societalactors	1	0	0.003334	-	-	-	-	-	-	1	0	0.00282	-	-	-
GERstate	Government	6	**553.292875**	0.033509	1	0	0.005586	**3**	36.5	0.006719	**3**	157.353968	0.044813	-	-	-
HAIcontractors	Forestindustry	**9**	287.981911	0.022799	**9**	**491.647028**	0.161081	-	-	-	2	42.509524	0.004378	-	-	-
HAIenvironmentalNGO	Societalactors	3	10.737996	0.004695	-	-	-	-	-	-	**3**	142.5	0.006281	-	-	-
HAIexpert	Societalactors	1	0	0.004507	-	-	-	-	-	-	1	0	0.011828	-	-	-
HAIforestauthority	Government	3	2.778388	0.008213	3	41.431349	0.06848	-	-	-	1	0	0	-	-	-
HAIforestcooperative	Forestowners	8	306.564964	0.032332	**7**	**394.825408**	0.17452	-	-	-	2	68.104762	0.01441	-	-	-

S4 Table continued.

		All nature's contributions to people			Timber			Climate regulation			Habitat creation and maintenance			Non-material nature's contributions to people		
HAIforester1	Foresters	7	**571.025961**	0.035094	3	225.572625	0.057734	1	0	0.007694	**3**	**301.247619**	0.051828	1	0	0
HAIforester10	Foresters	1	0	0.001441	-	-	-	-	-	-	1	0	0.001089	-	-	-
HAIforester2	Foresters	4	14.68028	0.007497	2	1.426445	0.059511	**2**	29	0.003062	1	0	0	1	0	0
HAIforester3	Foresters	8	304.848497	0.015113	**5**	120.006238	0.056154	**4**	**249**	0.07257	2	59	0.001512	-	-	-
HAIforester4	Foresters	1	0	0.004152	1	0	0.032651	-	-	-	-	-	-	-	-	-
HAIforester5	Foresters	7	188.335561	0.025746	**6**	305.063568	0.172154	-	-	-	1	0	0	1	0	0
HAIforester6	Foresters	8	311.301241	0.013941	3	11.169701	0.070136	**5**	**130**	0.012379	1	0	0	2	7	0
HAIforester7	Foresters	6	227.865138	0.030808	**6**	218.839998	0.113975	-	-	-	1	0	0	-	-	-
HAIforester8	Foresters	4	19.845435	0.005786	4	49.006238	0.054188	-	-	-	-	-	-	-	-	-
HAIforester9	Foresters	7	136.611892	0.011223	**6**	168.285502	0.102549	1	0	0.000999	**4**	150.080952	0.004771	2	15	0
HAIforesters	Foresters	3	12.421869	0.00664	3	13.245938	0.041973	-	-	-	-	-	-	-	-	-
HAIforestindustry	Forestindustry	2	2.742125	0.002684	2	0.5	0.020647	1	0	0.019835	-	-	-	-	-	-
HAIforestowners	Forestowners	**9**	196.758547	0.02335	**7**	115.590564	0.156985	**3**	77	0.010368	2	1	0	-	-	-
HAIgovernment	Government	3	8.111282	0.004073	2	0	0.026088	-	-	-	1	0	0.001089	2	16	0
HAInationalpark	Protectedareas	4	60.73935	0.006593	-	-	-	1	0	0.000837	2	87.314286	0.011661	1	0	0
HAInaturepark	Protectedareas	1	0	0.004507	-	-	-	-	-	-	-	-	-	1	0	0
HAIphilanthropy	Societalactors	1	0	0.00179	-	-	-	1	0	0.003384	-	-	-	-	-	-
HAIschool	Societalactors	1	0	0.001941	-	-	-	-	-	-	1	0	0.000345	-	-	-
HAIscience	Societalactors	1	0	0.00179	-	-	-	1	0	0.003384	1	0	0	-	-	-
HAIsociety	Societalactors	2	1.666667	0.003231	1	0	0.019188	**2**	29	0.003657	-	-	-	2	12	0
HAItourism	Societalactors	4	30.099813	0.005639	-	-	-	-	-	-	-	-	-	**4**	18	0
HAIworkers	Forestindustry	5	76.781381	0.012937	**5**	71.240443	0.0659	1	0	0.019835	-	-	-	-	-	-
SCHbiosphere reserve	Protectedareas	**15**	**606.531827**	0.105641	1	0	0.004198	**11**	**302**	**0.650825**	**7**	215.930664	0.153098	1	0	0
SCHcontractor1	Forestindustry	1	0	0.001949	1	0	0.00176	-	-	-	-	-	-	-	-	-
SCHcontractors	Forestindustry	**13**	384.865877	0.071663	**12**	**661.374413**	0.089966	1	0	0.177879	1	0	0.022273	-	-	-
SCHenvironmentalNGO	Societalactors	1	0	0.004016	-	-	-	-	-	-	1	0	0.011506	-	-	-
SCHexpert	Societalactors	1	0	0.006224	-	-	-	-	-	-	-	-	-	1	0	0
SCHforester1	Foresters	5	127.298967	0.049815	4	148.452381	0.022421	1	0	0.177879	**3**	57.3	0.093641	-	-	-
SCHforester10	Foresters	5	122.184373	0.031379	3	14.319603	0.035379	-	-	-	**3**	85.75873	0.055109	-	-	-
SCHforester11	Foresters	7	192.244303	0.048463	4	30.7363	0.050642	-	-	-	2	73.479798	0.048352	1	0	0
SCHforester12	Foresters	5	8.591182	0.034414	**5**	44.087055	0.061198	-	-	-	-	-	-	-	-	-
SCHforester13	Foresters	5	118.915197	0.027653	3	24.764168	0.03862	**2**	29	0.086838	-	-	-	-	-	-
SCHforester14	Foresters	4	101.214286	0.031273	2	62	0.022428	-	-	-	**3**	62.666667	0.050413	1	0	0
SCHforester15	Foresters	5	204.337121	0.019864	3	141	0.018112	-	-	-	2	59	0.012357	-	-	-
SCHforester16	Foresters	1	0	0.002551	1	0	0.003391	-	-	-	-	-	-	-	-	-
SCHforester2	Foresters	1	0	0.006397	1	0	0.004198	-	-	-	-	-	-	-	-	-
SCHforester4	Foresters	3	0.452381	0.024408	3	10.133333	0.03942	-	-	-	1	0	0.021471	-	-	-
SCHforester5	Foresters	8	290.425017	0.069695	4	91.589865	0.044991	-	-	-	**7**	**333.600216**	0.18254	2	2	0
SCHforester6	Foresters	6	214.535158	0.054034	2	73.809524	0.018602	**3**	29	**0.292089**	**3**	33.222222	0.104205	-	-	-
SCHforester7	Foresters	2	0.808362	0.01654	2	9.377375	0.032105	-	-	-	-	-	-	-	-	-
SCHforester8	Foresters	5	117.912698	0.025962	4	49.351892	0.052175	-	-	-	2	59	0.012357	-	-	-
SCHforester9	Foresters	6	242.068895	0.068714	3	**639.602856**	0.066912	-	-	-	**4**	244.133333	0.097591	-	-	-
SCHforesters	Foresters	**11**	397.475721	0.057136	**9**	369.820187	0.081528	1	0	0.052538	**4**	233.166667	0.051325	-	-	-
SCHforestindustry	Forestindustry	3	102.02291	0.015178	3	79.462283	0.009398	-	-	-	-	-	-	-	-	-
SCHforestlandusers	Forestindustry	1	0	0.013566	-	-	-	1	0	0.177879	-	-	-	-	-	-
SCHforestowners	Forestowners	8	387.345332	0.062207	**6**	194.053733	0.029838	**3**	56	**0.294004**	**3**	25.857143	0.056288	1	0	0
SCHforestryNGO	Forestindustry	1	0	0.002551	1	0	0.003391	-	-	-	-	-	-	-	-	-
SCHgovernment	Government	1	0	0.013566	-	-	-	1	0	0.177879	-	-	-	-	-	-
SCHphilanthropy	Societalactors	1	0	0.003551	-	-	-	1	0	0.023734	-	-	-	-	-	-
SCHtourism	Societalactors	2	0	0.017272	-	-	-	-	-	-	1	0	0.04166	1	0	0
SCHworkers	Forestindustry	5	30.974239	0.020775	4	2.208333	0.034793	-	-	-	1	0	0.00282	-	-	-
THstate	Government	8	214.857486	0.014858	3	6.053571	0.036881	**4**	**226.5**	0.028155	**3**	**252.42381**	0.014351	-	-	-
THstateforest	Government	6	62.634586	0.017644	**6**	98.877306	0.147867	-	-	-	-	-	-	1	0	0

S5 Table. Mean values and Chi-squares for degree, betweenness, and eigenvector centrality of actor groups contributing to the **overall NCP network and each focal NCP network** (timber production, climate regulation, creation and maintenance of habitat, non-material NCP). Bold values represent the actor group with the highest mean value for the respective centrality metric. Mean values with letters (a, b) indicate actor groups with significant differences to others. The level of significance is indicated for 1% (***), 5% (**), and 10% (*).

**Nature's contributions to people**	**Centrality metrics**	**Biodiversity Exploratories**	**Foresters**	**Forest industry**	**Forest owners**	**Government**	**Protected areas**	**Societal actors**	χ^2
All NCP	Degree	54.50^{a}	57.39^{b}	49.54^{a}	$\mathbf{77.13^{b}}$	61.50^{b}	64.25^{b}	30.84^{a}	19.70***
	Betweenness	$\mathbf{97.00^{ab}}$	58.13^{b}	45.54^{a}	85.50^{b}	57.87^{a}	61.50^{ab}	31.32^{a}	23.39***
	Eigenvector	64.00	55.61	47.35	63.75	59.37	54.38	39.44	7.15
Timber production	Degree	6.50	36.88	44.68	55.50	33.36	6.50	28.60	10.05
	Betweenness	7.00	39.59	39.09	50.50	30.14	7.00	33.60	7.91
	Eigenvector	8.00	37.78	36.50	47.50	36.93	4.50	36.40	5.26
Climate regulation	Degree	23.50^{a}	20.63^{a}	10.00^{a}	24.00^{a}	24.44^{a}	$\mathbf{26.33^{a}}$	11.93^{a}	11.20*
	Betweenness	35.00	21.17	10.50	25.17	21.25	24.50	13.00	10.35
	Eigenvector	34.00	16.96	24.50	21.00	21.75	16.67	16.93	4.13
Creation and maintenance of habitat	Degree	44.00^{ab}	33.62^{a}	16.50^{a}	37.75^{ab}	45.27^{b}	$\mathbf{53.17^{b}}$	29.69^{a}	13.00**
	Betweenness	51.00^{ab}	34.20^{a}	17.33^{a}	37.50^{ab}	45.64^{b}	$\mathbf{54.00^{b}}$	27.00^{a}	14.50**
	Eigenvector	40.00	32.93	27.67	29.75	41.59	45.33	33.81	3.55
Non-material NCP	Degree	10.50	20.75	10.50	10.50	23.57	17.00	23.14	5.02
	Betweenness	10.50	20.75	10.50	10.50	24.36	15.88	23.05	5.53
	Eigenvector	24.50	18.36	27.50	11.00	23.43	18.00	21.50	3.12

S6 Table. Mean values and Chi-squares for degree, betweenness, and eigenvector centrality of actor groups regarding their use of the anthropogenic capitals contributing to the co-production **timber**. Bold values represent the actor group with the highest mean value for the respective centrality metric. Mean values with letters (a, b) indicate actor groups with significant differences to others. The level of significance is indicated for 1% (***), 5% (**), and 10% (*).

**Anthropo-genic capitals**	**Centrality metrics**	**Biodiversity Exploratories**	**Foresters**	**Forest industry**	**Forest owners**	**Government**	**Protected areas**	**Societal actors**	χ^2

Human capital	Degree	8.00	33.22	42.33	28.33	23.50	8.00	21.75	10.23
	Betweenness	9.00	34.56	37.11	32.00	20.38	9.00	24.50	8.84
	Eigenvector	5.50	33.53	33.89	11.83	34.94	7.50	28.25	8.45
Social capital	Degree	-	28.76	22.50	39.38	34.27	14.50	22.80	6.59
	Betweenness	-	30.54	20.72	36.38	32.92	14.50	23.00	6.11
	Eigenvector	-	28.26	23.00	32.88	35.85	8.50	26.70	5.40
Physical capital	Degree	-	24.77	34.00	24.00	22.11	-	37.00	5.23
	Betweenness	-	26.30	31.70	26.50	18.78	-	38.00	5.12
	Eigenvector	-	25.33	25.70	27.00	27.56	-	40.50	2.06
Financial capital	Degree	-	10.60^{a}	10.75^{ab}	**21.50^{b}**	14.50^{ab}	-	-	7.79*
	Betweenness	-	11.70^{a}	11.75^{ab}	**21.38^{b}**	12.689^{ab}	-	-	7.07*
	Eigenvector	-	13.00	11.00	16.50	13.88	-	-	1.12

S7 Table. Mean values and Chi-squares for degree, betweenness, and eigenvector centrality of actor groups regarding their use of the anthropogenic capitals contributing to the **regulation of climate**. No analysis was performed for physical capital contributing to the co-production of this NCP as too few connections were found. Bold values represent the actor group with the highest mean value for the respective centrality metric. Mean values with letters (a, b) indicate actor groups with significant differences to others. The level of significance is indicated for 1% (***), 5% (**), and 10% (*).

**Anthropo-genic capitals**	**Centrality metrics**	**Biodiversity Exploratories**	**Foresters**	**Forest industry**	**Forest owners**	**Government**	**Protected areas**	**Societal actors**	χ^2
Human capital	Degree	5.00	8.60	5.00	-	-	-	5.00	5.04
	Betweenness	5.00	8.60	5.00	-	-	-	5.00	5.04
	Eigenvector	**10.50^{a}**	5.70^{a}	**10.50^{a}**	-	-	-	4.50^{a}	7.70*
Social capital	Degree	-	8.14	7.00	11.00	9.00	14.00	7.00	5.18
	Betweenness	-	8.00	7.00	11.75	9.38	13.33	7.00	4.65
	Eigenvector	-	9.00	13.50	10.75	10.00	9.33	5.00	1.72
Physical capital	Degree	-	-	-	-	-	-	-	-
	Betweenness	-	-	-	-	-	-	-	-
	Eigenvector	-	-	-	-	-	-	-	-
Financial capital	Degree	-	7.68	-	8.33	12.50	13.00	6.00	5.28
	Betweenness	-	7.83	-	8.50	12.17	13.50	6.00	4.67
	Eigenvector	-	10.25	-	11.17	9.00	5.50	8.25	1.37

S8 Table. Mean values and Chi-squares for degree, betweenness, and eigenvector centrality of actor groups regarding their use of the anthropogenic capitals contributing to the **creation and maintenance of habitat**. Bold values represent the actor group with the highest mean value for the respective centrality metric. Mean values with letters (a, b) indicate actor groups with significant differences to others. The level of significance is indicated for 1% (***), 5% (**), and 10% (*).

Anthropo-genic capitals	Centrality metrics	Biodiversity Exploratories	Foresters	Forest industry	Forest owners	Government	Protected areas	Societal actors	χ^2
Human capital	Degree	31.00	15.94	13.00	20.25	20.25	-	18.89	5.27
	Betweenness	30.00	15.67	13.00	20.75	22.50	-	18.94	5.81
	Eigenvector	24.00	14.67	15.00	16.00	28.50	-	20.89	6.71
Social capital	Degree	-	16.18[a]	10.00[a]	10.00[a]	14.07[a]	**26.17[a]**	10.00[a]	11.48**
	Betweenness	-	15.86[a]	10.00[a]	10.00[a]	14.86[a]	**25.50[a]**	10.00[a]	10.37*
	Eigenvector	-	18.86	12.00	6.50	12.14	16.50	14.00	5.87
Physical capital	Degree	-	11.42	10.50	10.50	10.50		12.70	1.17
	Betweenness	-	11.42	10.50	10.50	10.50		12.70	1.17
	Eigenvector	-	11.33	9.50	9.50	9.50		13.90	2.58
Financial capital	Degree		13.96	9.00	20.50	19.61	9.00	18.67	5.66
	Betweenness		15.18	9.00	18.50	18.06	9.00	19.00	3.39
	Eigenvector		17.29	13.75	17.50	16.06	9.00	12.68	1.74

S9 Table. Mean values and Chi-squares for degree, betweenness, and eigenvector centrality of actor groups regarding their use of the anthropogenic capitals contributing to the co-production of **non-material NCP**. Bold values represent the actor group with the highest mean value for the respective centrality metric. Mean values with letters (a, b) indicate actor groups with significant differences to others. The level of significance is indicated for 1% (***), 5% (**), and 10% (*).

Anthropo-genic capitals	Centrality metrics	Biodiversity Exploratories	Foresters	Forest industry	Forest owners	Government	Protected areas	Societal actors	χ^2
Human capital	Degree	9.00	14.33	9.00	9.00	19.67	20.00	11.60	6.22
	Betweenness	9.00	14.22	9.00	9.00	19.67	21.00	11.60	6.46
	Eigenvector	19.50	11.33	15.00	7.50	18.50	21.00	13.05	5.06
Social	Degree	-	13.00	6.00	-	13.40	10.38	13.58	1.656

capital	Betweenness	-	11.81	6.50	-	14.80	10.38	13.92	2.20
	Eigenvector	-	12.06	16.00	-	15.70	9.00	12.17	2.32
Physical capital	Degree	-	10.61	9.50	9.50	9.50	9.50	15.17	6.08
	Betweenness	-	10.61	9.50	9.50	9.50	9.50	15.17	6.08
	Eigenvector	-	10.17	6.50	6.50	16.83	10.00	13.00	4.83
Financial capital	Degree	-	6.30	-	-	9.50	11.50	9.00	2.20
	Betweenness	-	5.50	-	-	10.08	11.00	9.25	3.99
	Eigenvector	-	8.00	-	-	9.08	9.00	8.13	0.19
